# Supplementary material for: A Humanized Yeast Phenomic Model of Deoxycytidine Kinase to Predict Genetic Buffering of Nucleoside Analog Cytotoxicity
Source: Genes (Basel). 2019 Sep 30;10(10):770. doi: 10.3390/genes10100770 (PMC6826991; doi:10.3390/genes10100770)
Supplement: Supplementary file 1 [file genes-10-00770-s001.zip › Additional_Files/Additional_File7_GOTermHeatmaps.zip!/Additional_File7_GOTermHeatmaps/covalent_chromatin_modification.pdf]

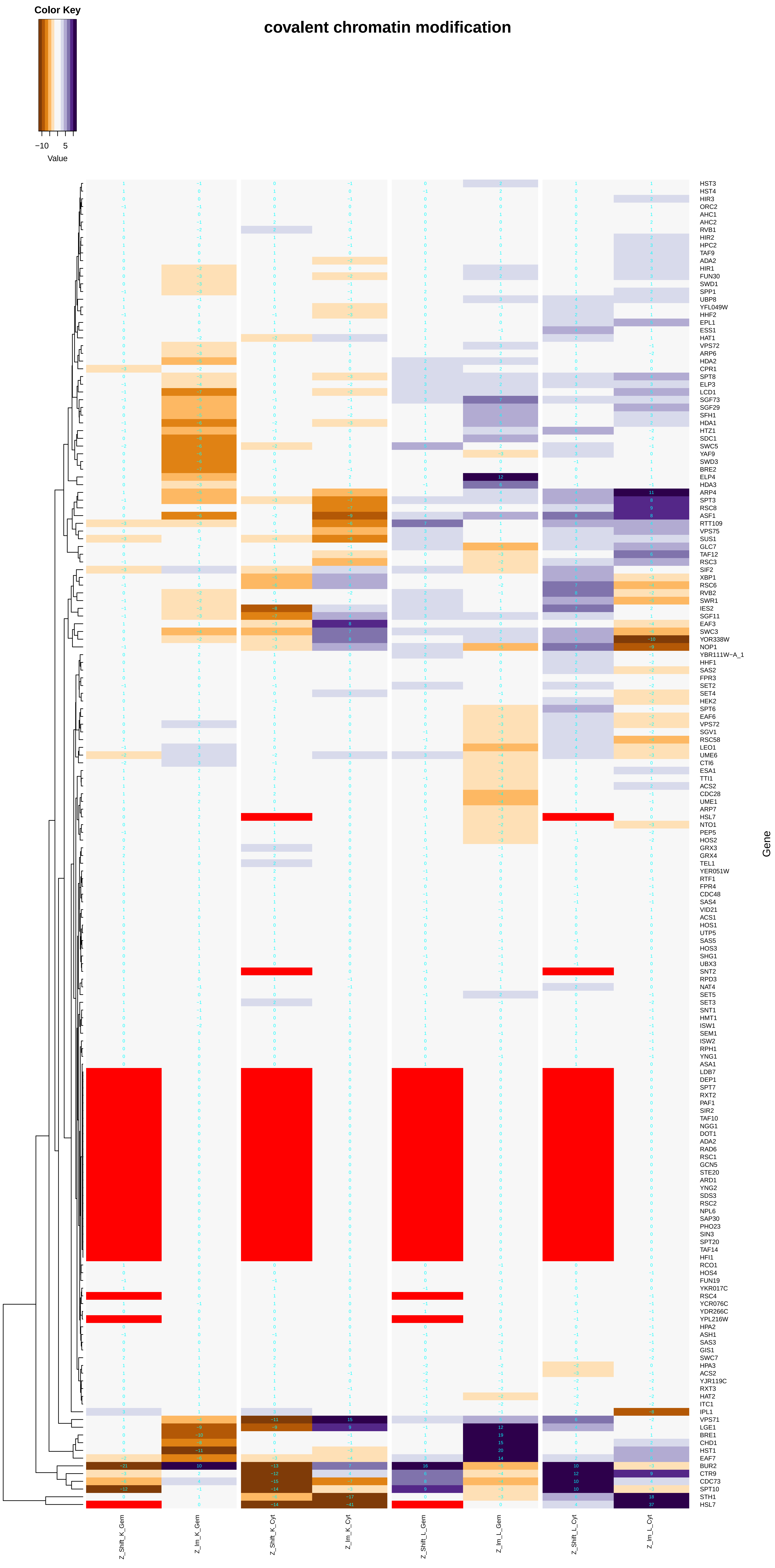
Color Key
−10
5
Value
covalent chromatin modification
Gene
Z_Shift_K_Gem
Z_Im_K_Gem
Z_Shift_K_Cyt
Z_Im_K_Cyt
Z_Shift_L_Gem
Z_Im_L_Gem
Z_Shift_L_Cyt
Z_Im_L_Cyt

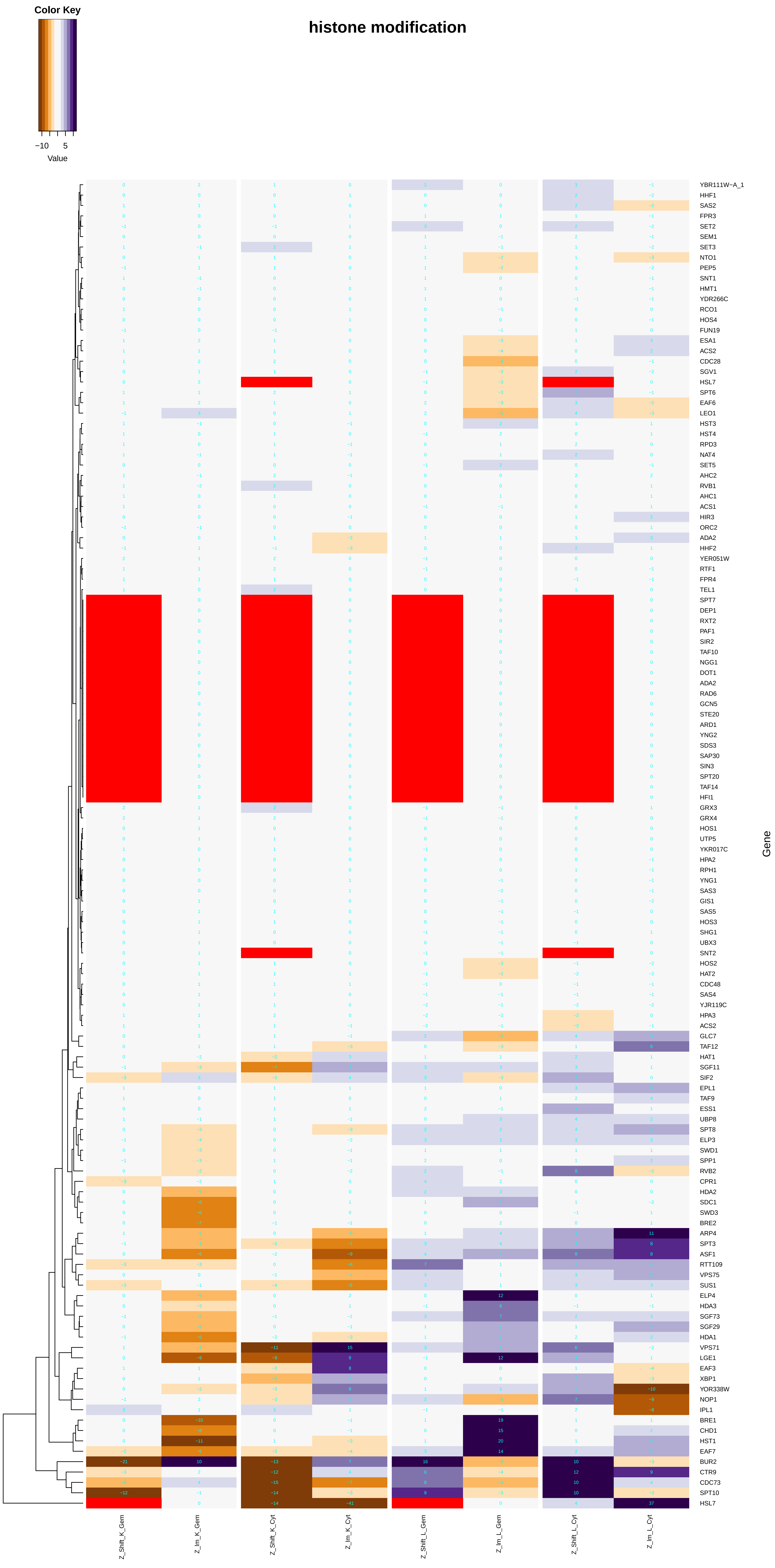

Color Key
−10
5
Value
histone modification
Z_Shift_K_Gem
Z_Im_K_Gem
Z_Shift_K_Cyt
Z_Im_K_Cyt
Z_Shift_L_Gem
Z_Im_L_Gem
Z_Shift_L_Cyt
Z_Im_L_Cyt
Gene
YBR111W−A_1
HHF1
SAS2
FPR3
SET2
SEM1
SET3
NTO1
PEP5
SNT1
HMT1
YDR266C
RCO1
HOS4
FUN19
ESA1
ACS2
CDC28
SGV1
HSL7
SPT6
EAF6
LEO1
HST3
HST4
RPD3
NAT4
SET5
AHC2
RVB1
AHC1
ACS1
HIR3
ORC2
ADA2
HHF2
YER051W
RTF1
FPR4
TEL1
SPT7
DEP1
RXT2
PAF1
SIR2
TAF10
NGG1
DOT1
ADA2
RAD6
GCN5
STE20
ARD1
YNG2
SDS3
SAP30
SIN3
SPT20
TAF14
HFI1
GRX3
GRX4
HOS1
UTP5
YKR017C
HPA2
RPH1
YNG1
SAS3
GIS1
SAS5
HOS3
SHG1
UBX3
SNT2
HOS2
HAT2
CDC48
SAS4
YJR119C
HPA3
ACS2
GLC7
TAF12
HAT1
SGF11
SIF2
EPL1
TAF9
ESS1
UBP8
SPT8
ELP3
SWD1
SPP1
RVB2
CPR1
HDA2
SDC1
SWD3
BRE2
ARP4
SPT3
ASF1
RTT109
VPS75
SUS1
ELP4
HDA3
SGF73
SGF29
HDA1
VPS71
LGE1
EAF3
XBP1
YOR338W
NOP1
IPL1
BRE1
CHD1
HST1
EAF7
BUR2
CTR9
CDC73
SPT10
HSL7

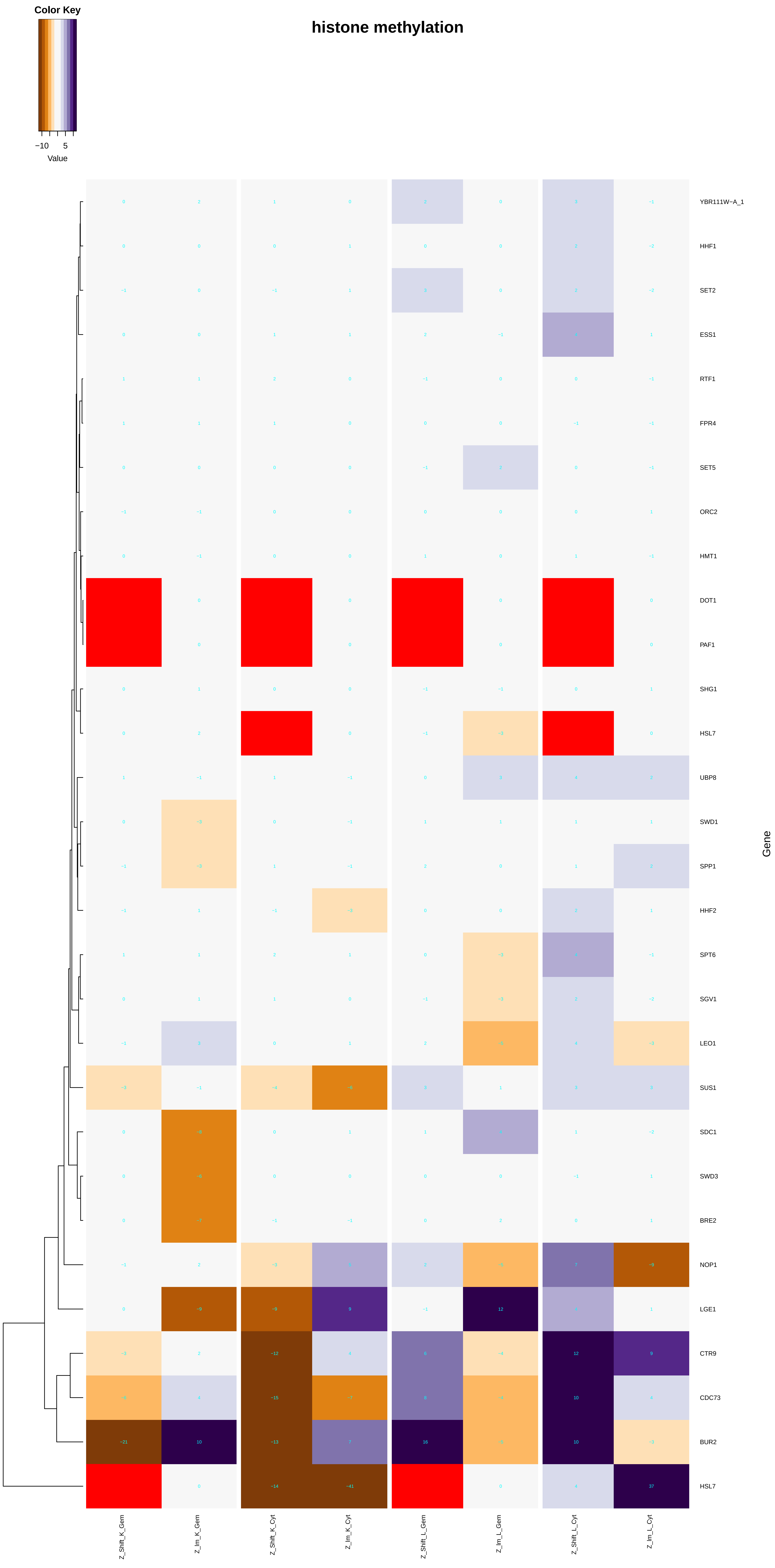
histone methylation
Color Key
−10
5
Value
YBR111W−A_1
HHF1
SET2
ESS1
RTF1
FPR4
SET5
ORC2
HMT1
DOT1
PAF1
SHG1
HSL7
UBP8
SWD1
SPP1
HHF2
SPT6
SGV1
LEO1
SUS1
SDC1
SWD3
BRE2
NOP1
LGE1
CTR9
CDC73
BUR2
HSL7
Gene
Z_Shift_K_Gem
Z_Im_K_Gem
Z_Shift_K_Cyt
Z_Im_K_Cyt
Z_Shift_L_Gem
Z_Im_L_Gem
Z_Shift_L_Cyt
Z_Im_L_Cyt

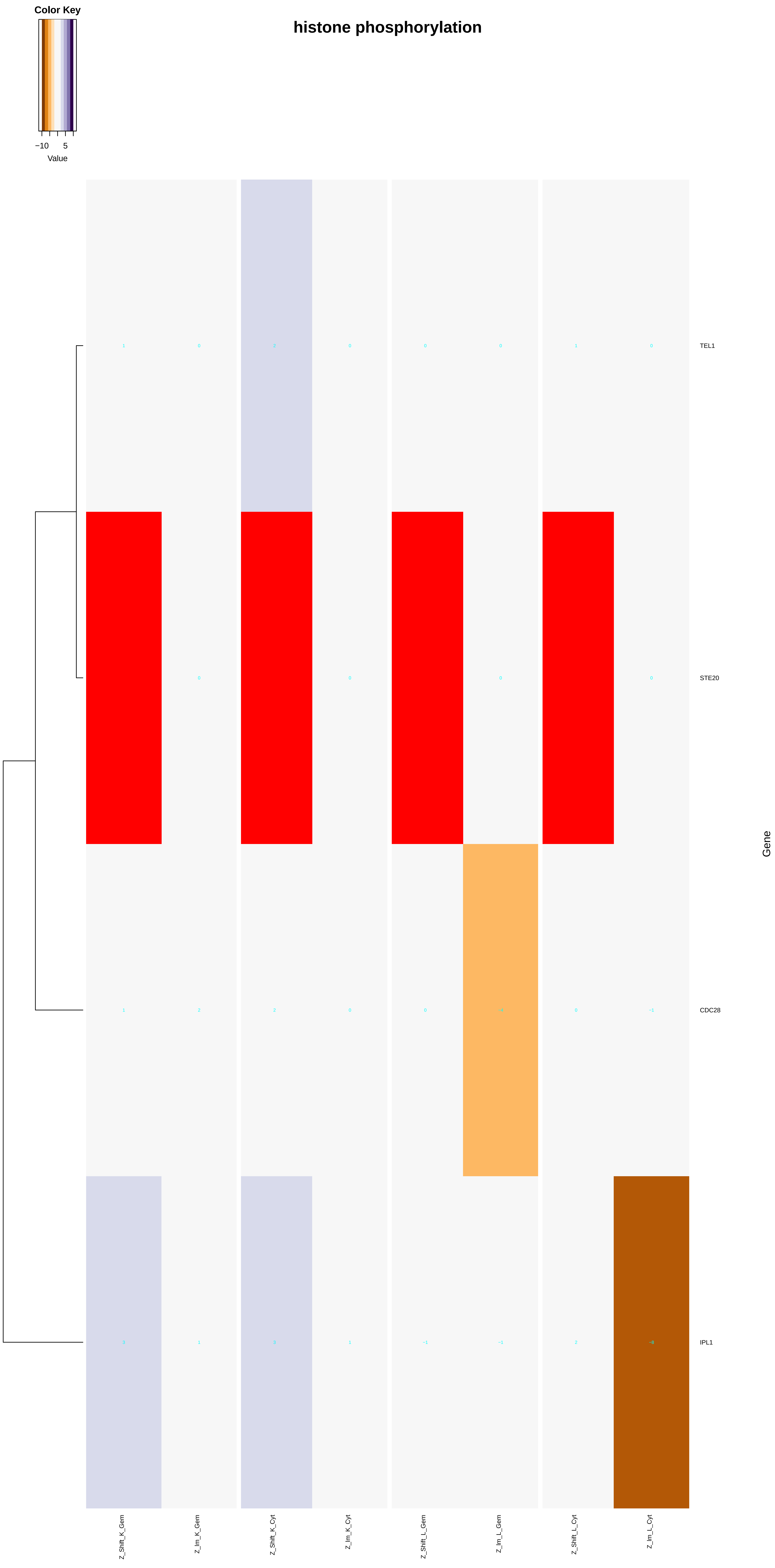

Color Key
−10
5
Value
histone phosphorylation
TEL1
STE20
CDC28
IPL1
Gene
Z_Shift_K_Gem
Z_Im_K_Gem
Z_Shift_K_Cyt
Z_Im_K_Cyt
Z_Shift_L_Gem
Z_Im_L_Gem
Z_Shift_L_Cyt
Z_Im_L_Cyt

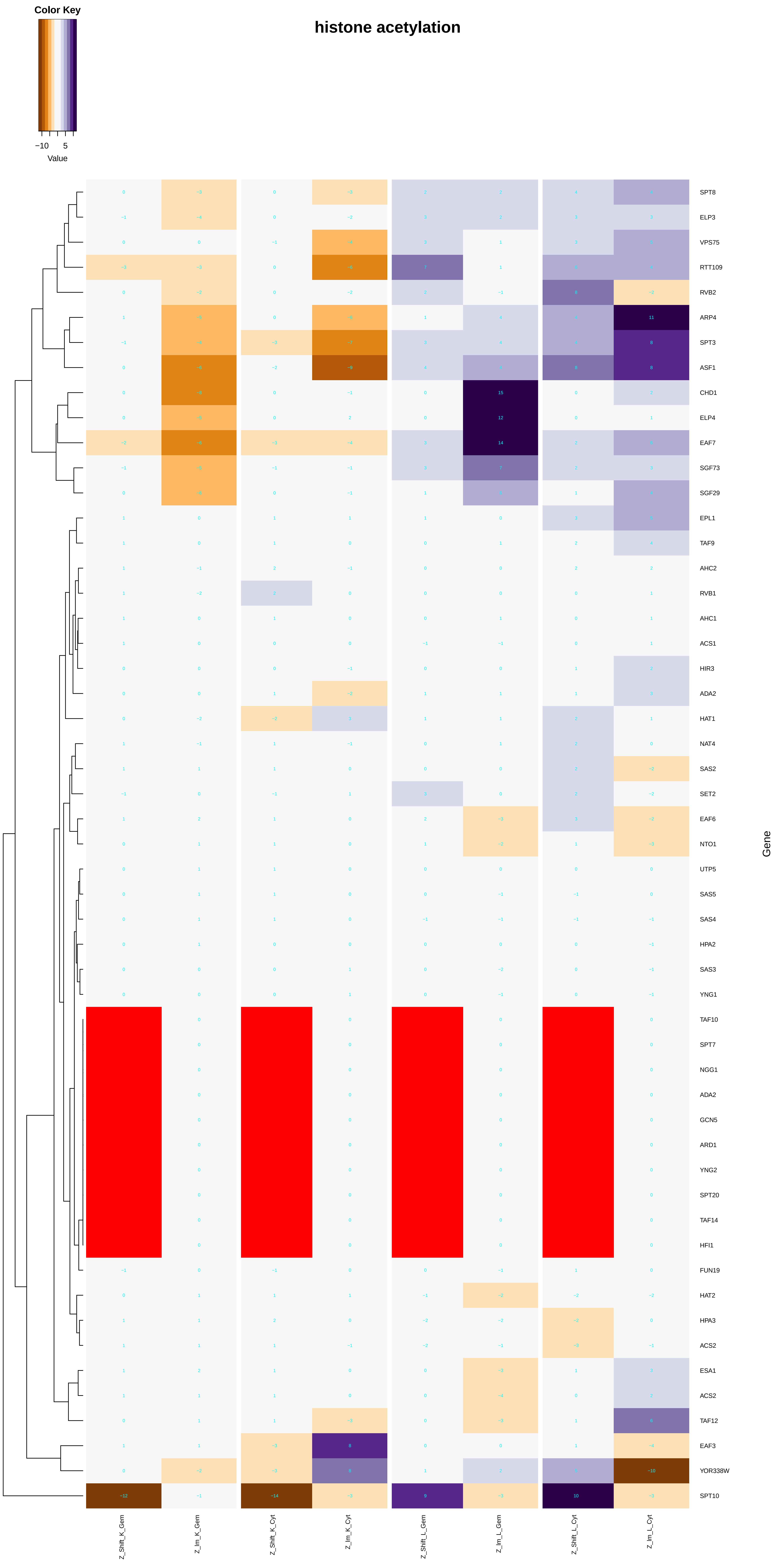
histone acetylation
Color Key
−10
5
Value
SPT8
ELP3
VPS75
RTT109
RVB2
ARP4
SPT3
ASF1
CHD1
ELP4
EAF7
SGF73
SGF29
EPL1
TAF9
AHC2
RVB1
AHC1
ACS1
HIR3
ADA2
HAT1
NAT4
SAS2
SET2
EAF6
NTO1
UTP5
SAS5
SAS4
HPA2
SAS3
YNG1
TAF10
SPT7
NGG1
ADA2
GCN5
ARD1
YNG2
SPT20
TAF14
HFI1
FUN19
HAT2
HPA3
ACS2
ESA1
ACS2
TAF12
EAF3
YOR338W
SPT10
Gene
Z_Shift_K_Gem
Z_Im_K_Gem
Z_Shift_K_Cyt
Z_Im_K_Cyt
Z_Shift_L_Gem
Z_Im_L_Gem
Z_Shift_L_Cyt
Z_Im_L_Cyt

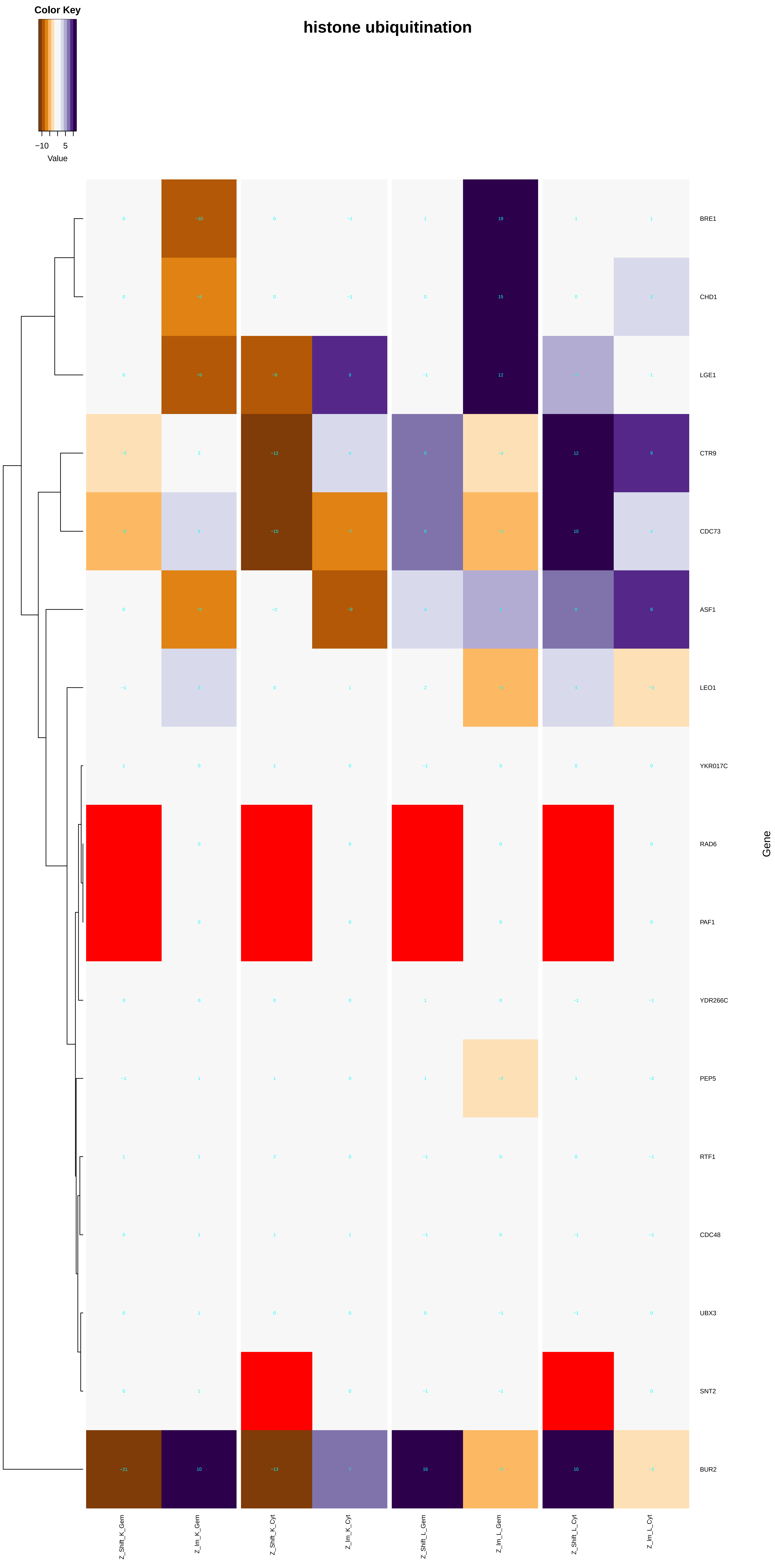
Color Key
−10
5
Value
histone ubiquitination
BRE1
CHD1
LGE1
CTR9
CDC73
ASF1
LEO1
YKR017C
RAD6
PAF1
YDR266C
PEP5
RTF1
CDC48
UBX3
SNT2
BUR2
Gene
Z_Shift_K_Gem
Z_Im_K_Gem
Z_Shift_K_Cyt
Z_Im_K_Cyt
Z_Shift_L_Gem
Z_Im_L_Gem
Z_Shift_L_Cyt
Z_Im_L_Cyt

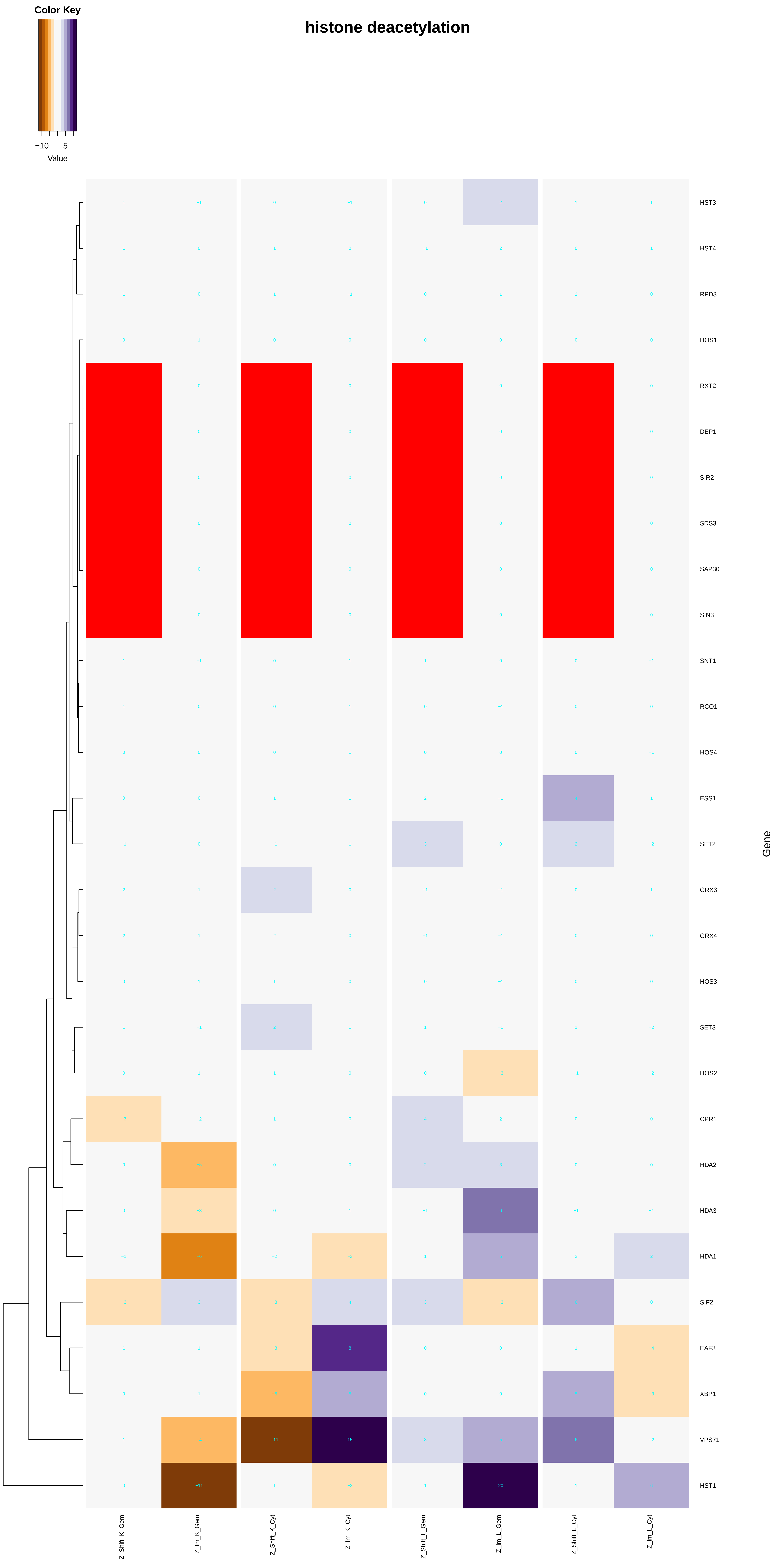
Color Key
−10 5
Value
histone deacetylation
HST3
HST4
RPD3
HOS1
RXT2
DEP1
SIR2
SDS3
SAP30
SIN3
SNT1
RCO1
HOS4
ESS1
SET2
GRX3
GRX4
HOS3
SET3
HOS2
CPR1
HDA2
HDA3
HDA1
SIF2
EAF3
XBP1
VPS71
HST1
Gene
Z_Shift_K_Gem
Z_Im_K_Gem
Z_Shift_K_Cyt
Z_Im_K_Cyt
Z_Shift_L_Gem
Z_Im_L_Gem
Z_Shift_L_Cyt
Z_Im_L_Cyt

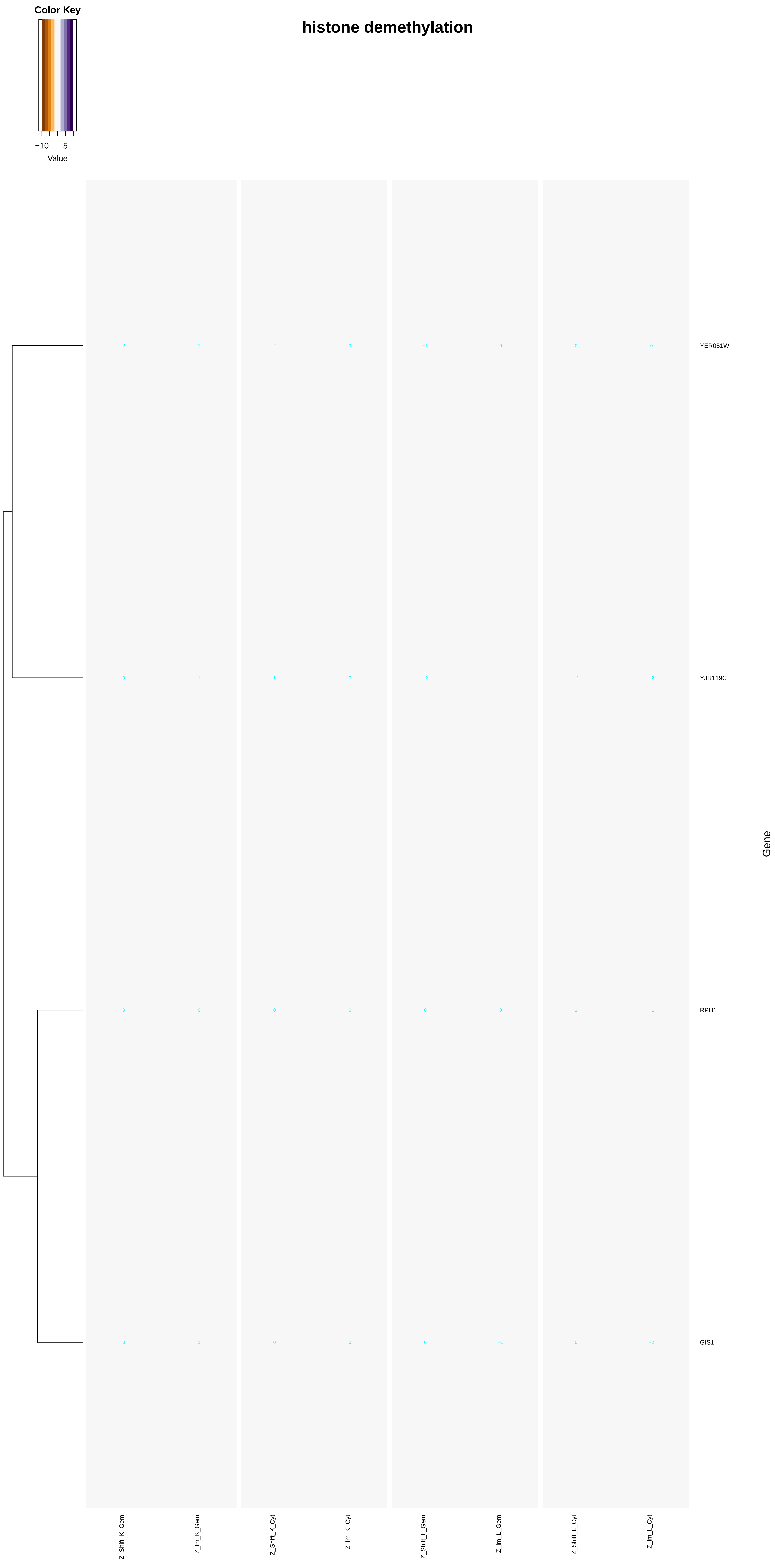
Color Key
−10
5
Value
histone demethylation
YER051W
YJR119C
RPH1
GIS1
Gene
Z_Shift_K_Gem
Z_Im_K_Gem
Z_Shift_K_Cyt
Z_Im_K_Cyt
Z_Shift_L_Gem
Z_Im_L_Gem
Z_Shift_L_Cyt
Z_Im_L_Cyt

histone deubiquitination

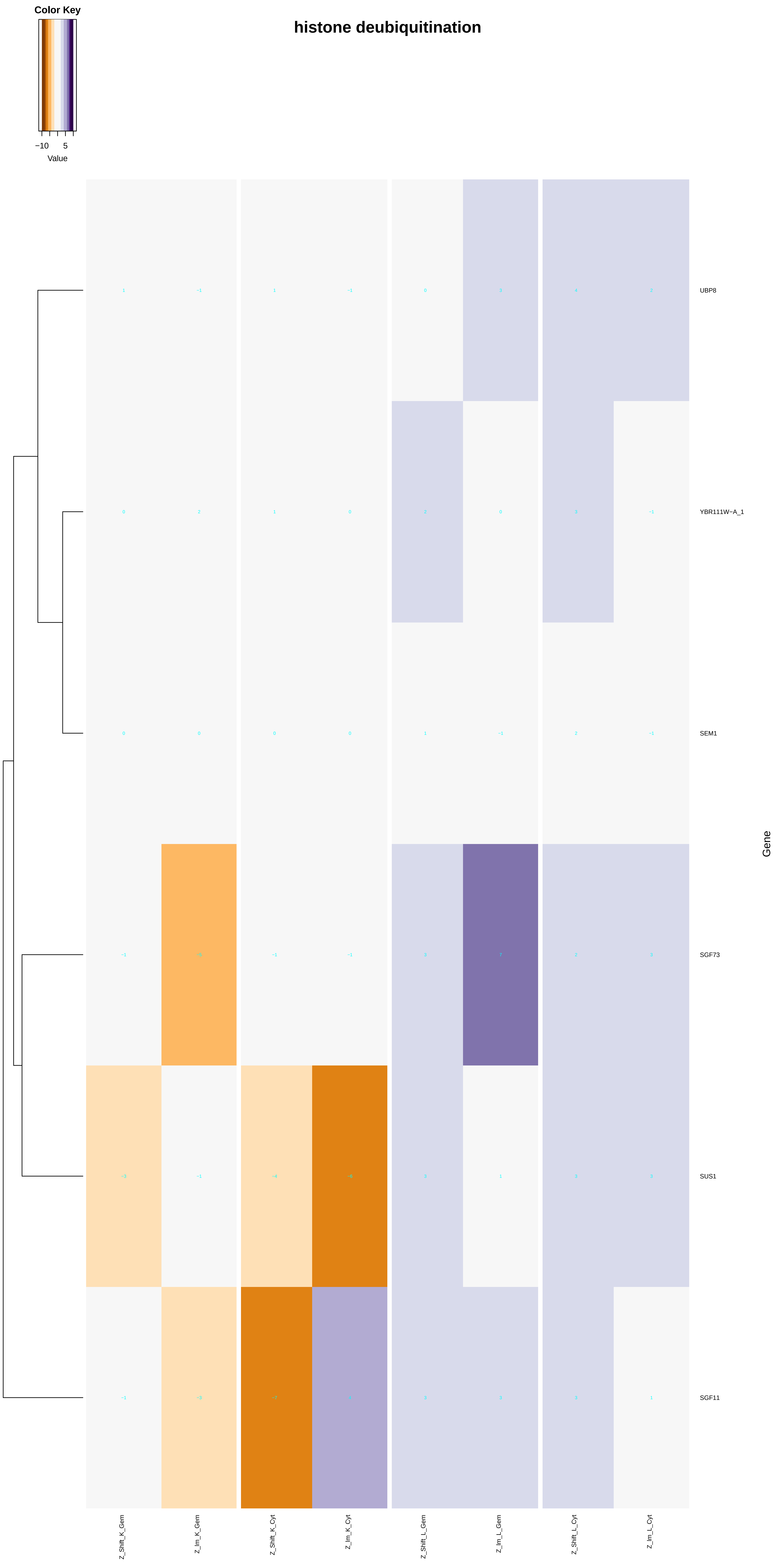

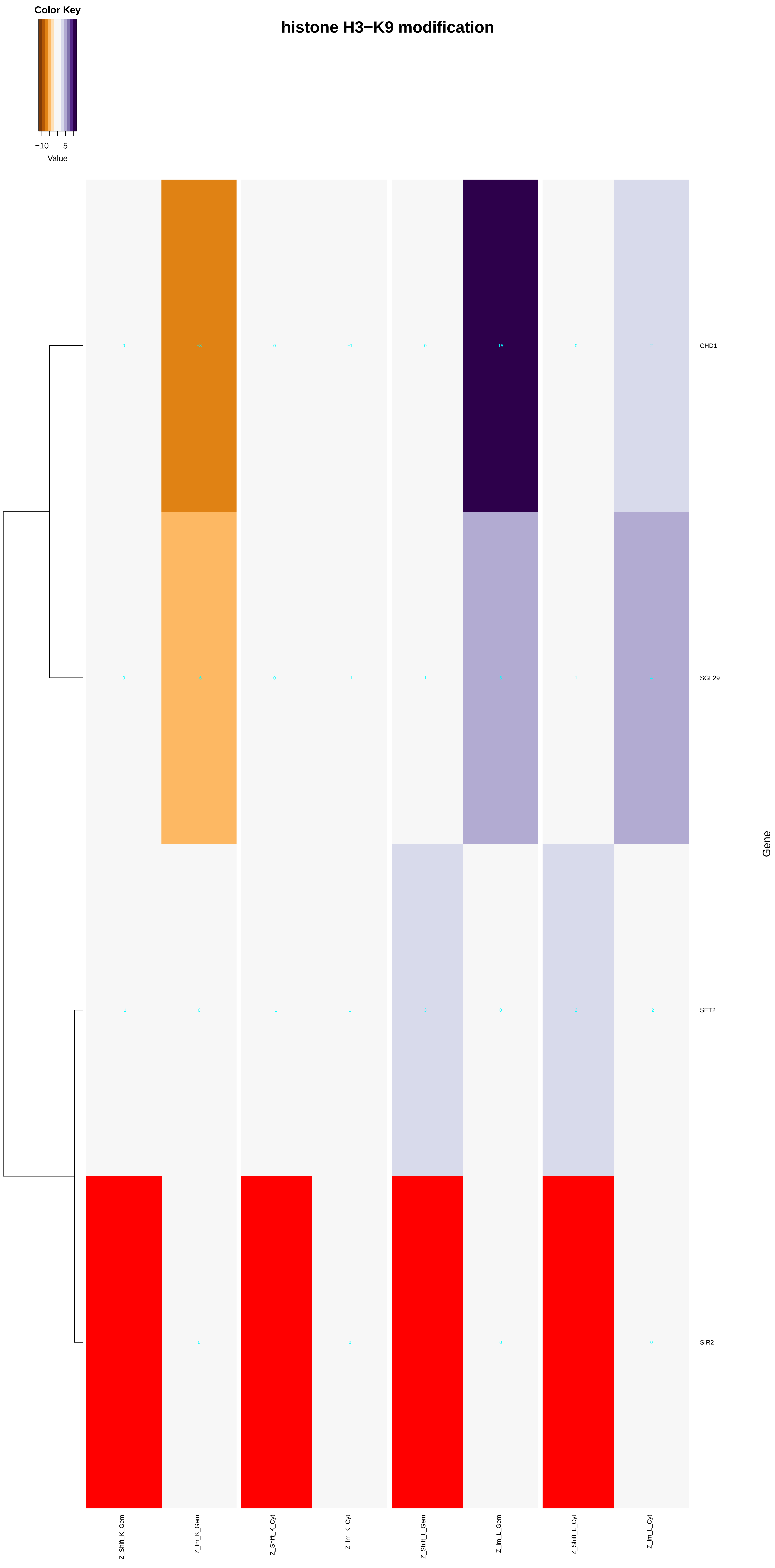
histone H3−K9 modification
Color Key
−10
5
Value
CHD1
SGF29
SET2
SIR2
Gene
Z_Shift_K_Gem
Z_Im_K_Gem
Z_Shift_K_Cyt
Z_Im_K_Cyt
Z_Shift_L_Gem
Z_Im_L_Gem
Z_Shift_L_Cyt
Z_Im_L_Cyt

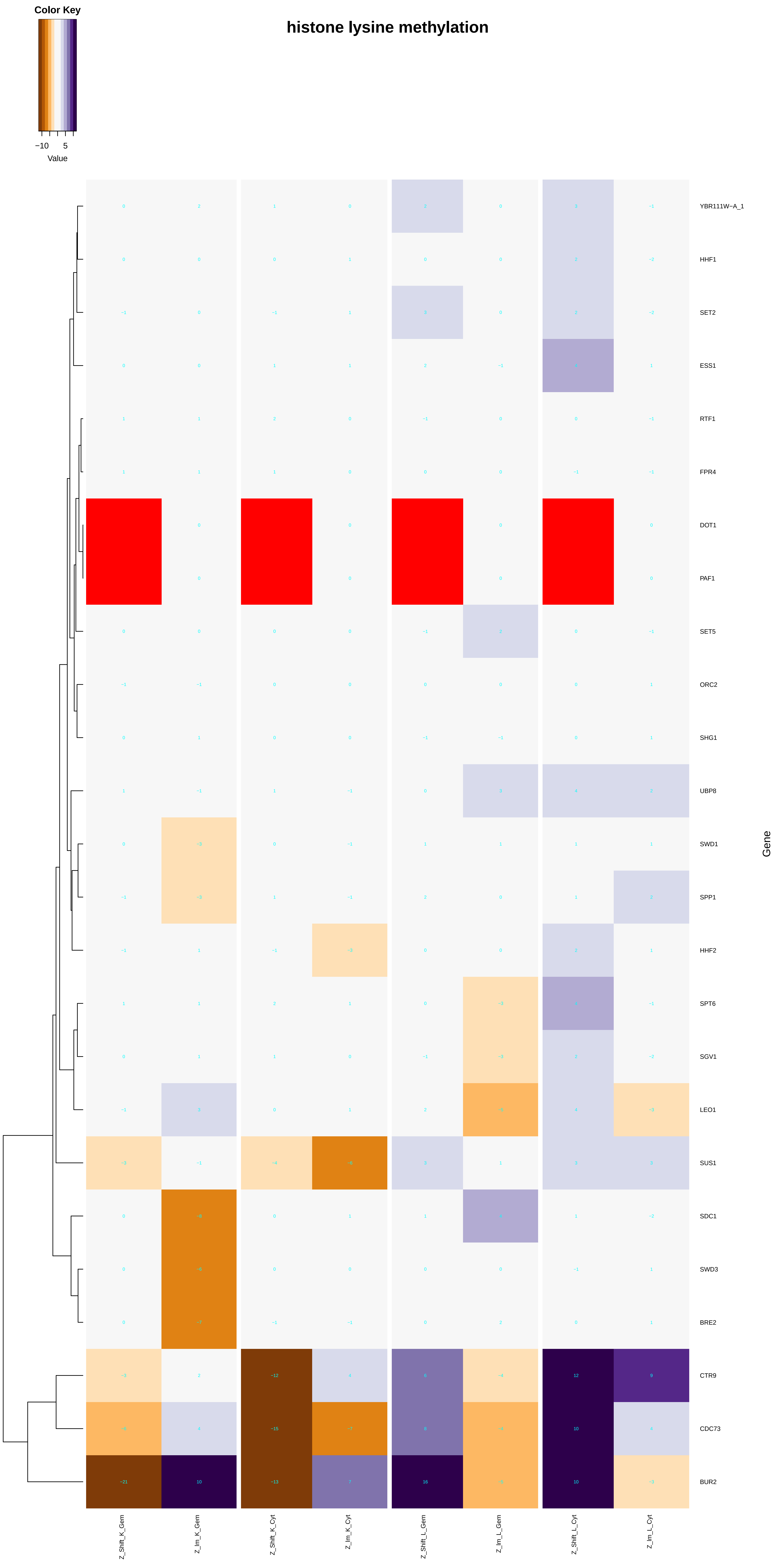

histone lysine methylation
Color Key
−10
5
Value
YBR111W−A_1
HHF1
SET2
ESS1
RTF1
FPR4
DOT1
PAF1
SET5
ORC2
SHG1
UBP8
SWD1
SPP1
HHF2
SPT6
SGV1
LEO1
SUS1
SDC1
SWD3
BRE2
CTR9
CDC73
BUR2
Gene
Z_Shift_K_Gem
Z_Im_K_Gem
Z_Shift_K_Cyt
Z_Im_K_Cyt
Z_Shift_L_Gem
Z_Im_L_Gem
Z_Shift_L_Cyt
Z_Im_L_Cyt

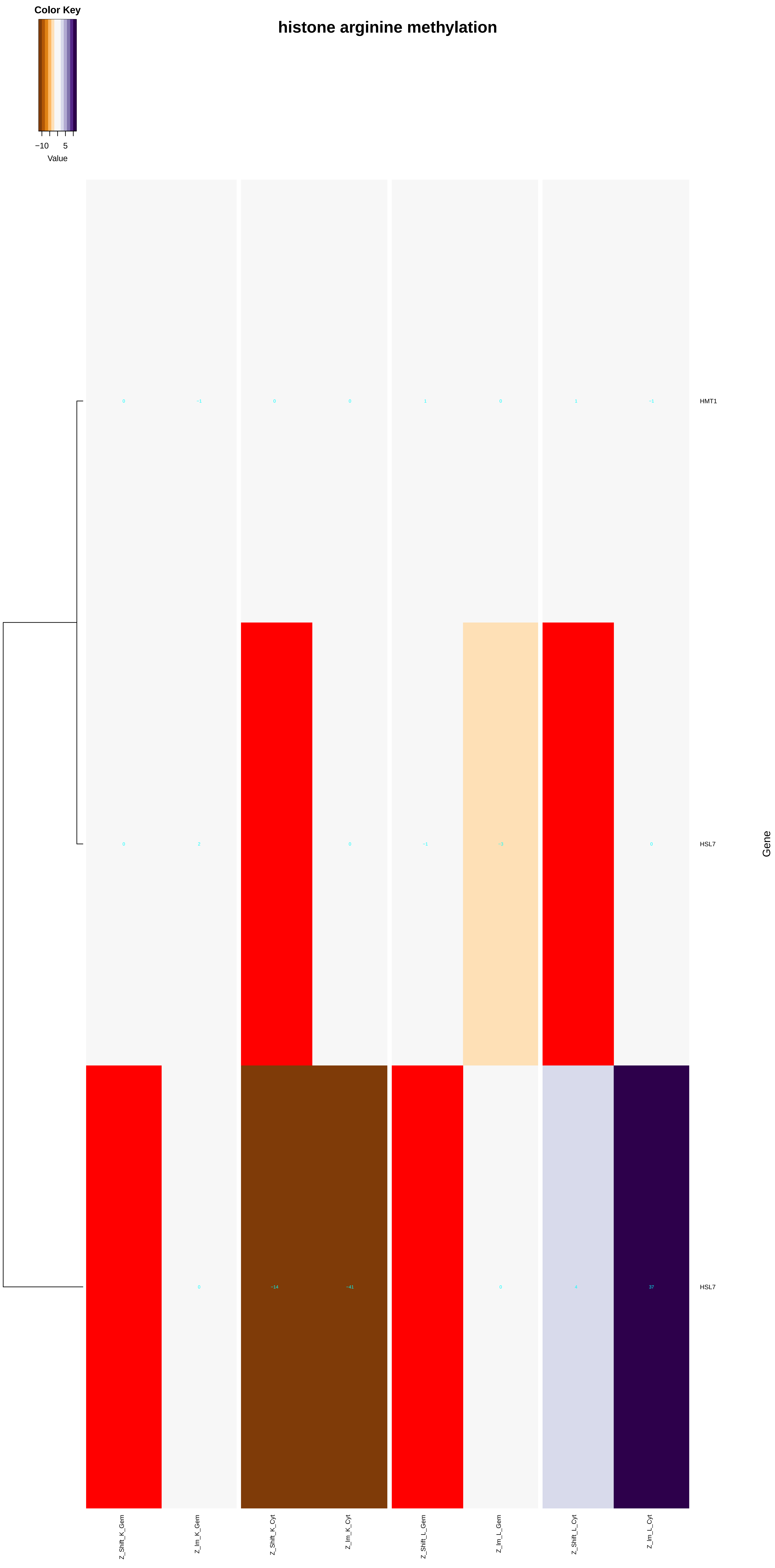
Color Key
−10
5
Value
histone arginine methylation
HMT1
HSL7
HSL7
Gene
Z_Shift_K_Gem
Z_Im_K_Gem
Z_Shift_K_Cyt
Z_Im_K_Cyt
Z_Shift_L_Gem
Z_Im_L_Gem
Z_Shift_L_Cyt
Z_Im_L_Cyt

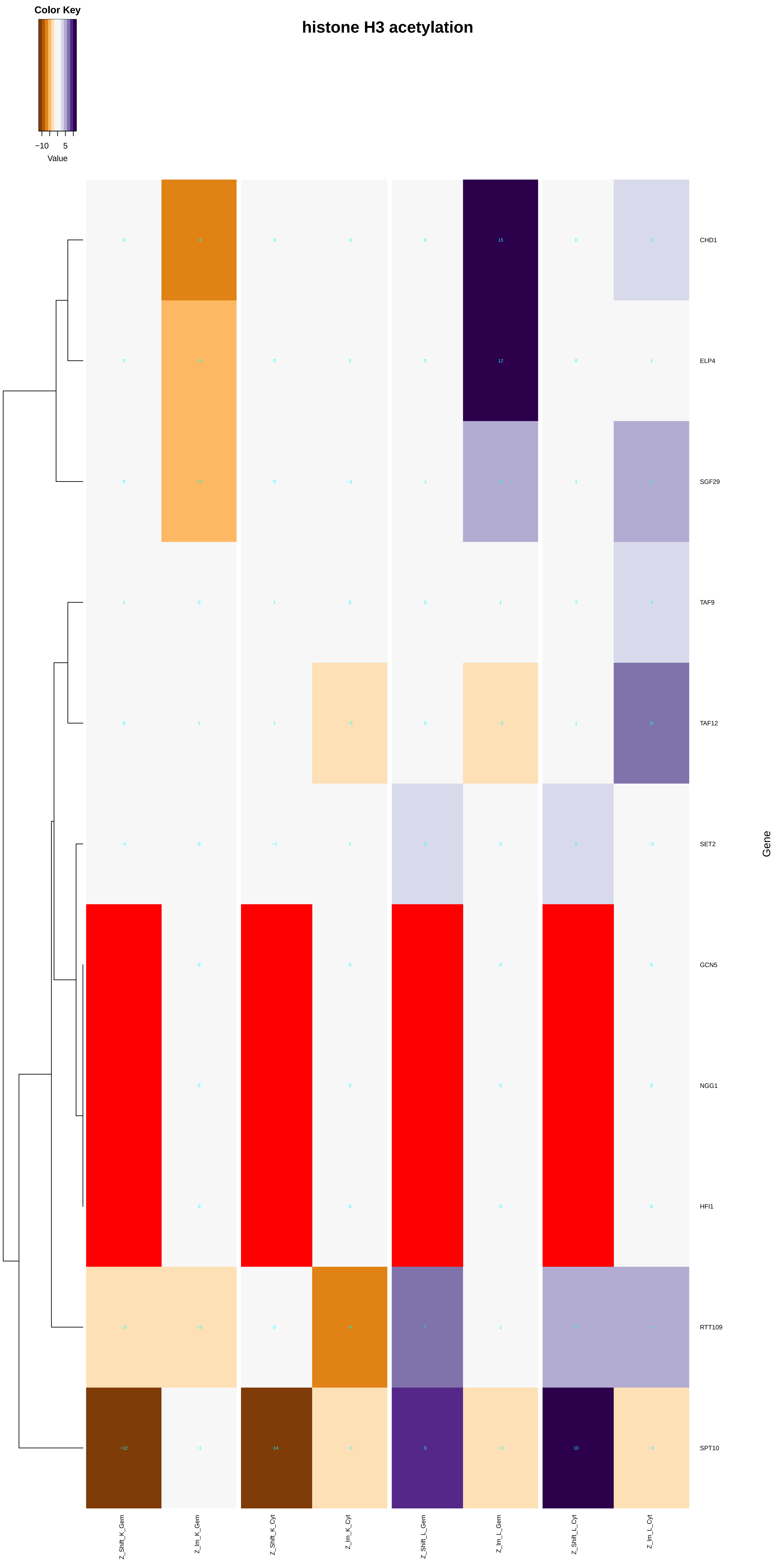
histone H3 acetylation
Color Key
−10
5
Value
CHD1
ELP4
SGF29
TAF9
TAF12
SET2
GCN5
NGG1
HFI1
RTT109
SPT10
Gene
Z_Shift_K_Gem
Z_Im_K_Gem
Z_Shift_K_Cyt
Z_Im_K_Cyt
Z_Shift_L_Gem
Z_Im_L_Gem
Z_Shift_L_Cyt
Z_Im_L_Cyt

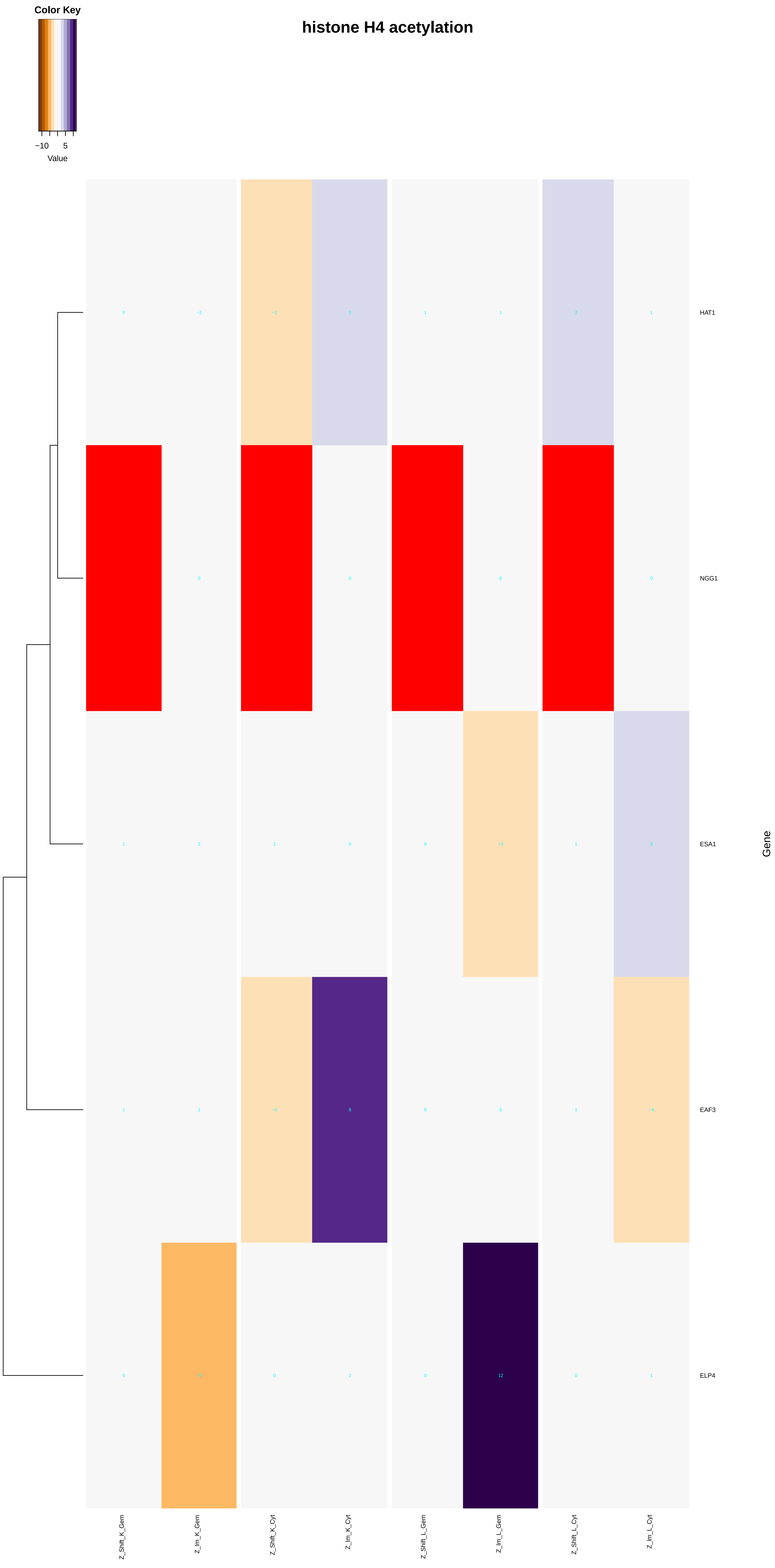

histone H4 acetylation
Color Key
−10
5
Value
HAT1
NGG1
ESA1
EAF3
ELP4
Gene
Z_Shift_K_Gem
Z_Im_K_Gem
Z_Shift_K_Cyt
Z_Im_K_Cyt
Z_Shift_L_Gem
Z_Im_L_Gem
Z_Shift_L_Cyt
Z_Im_L_Cyt

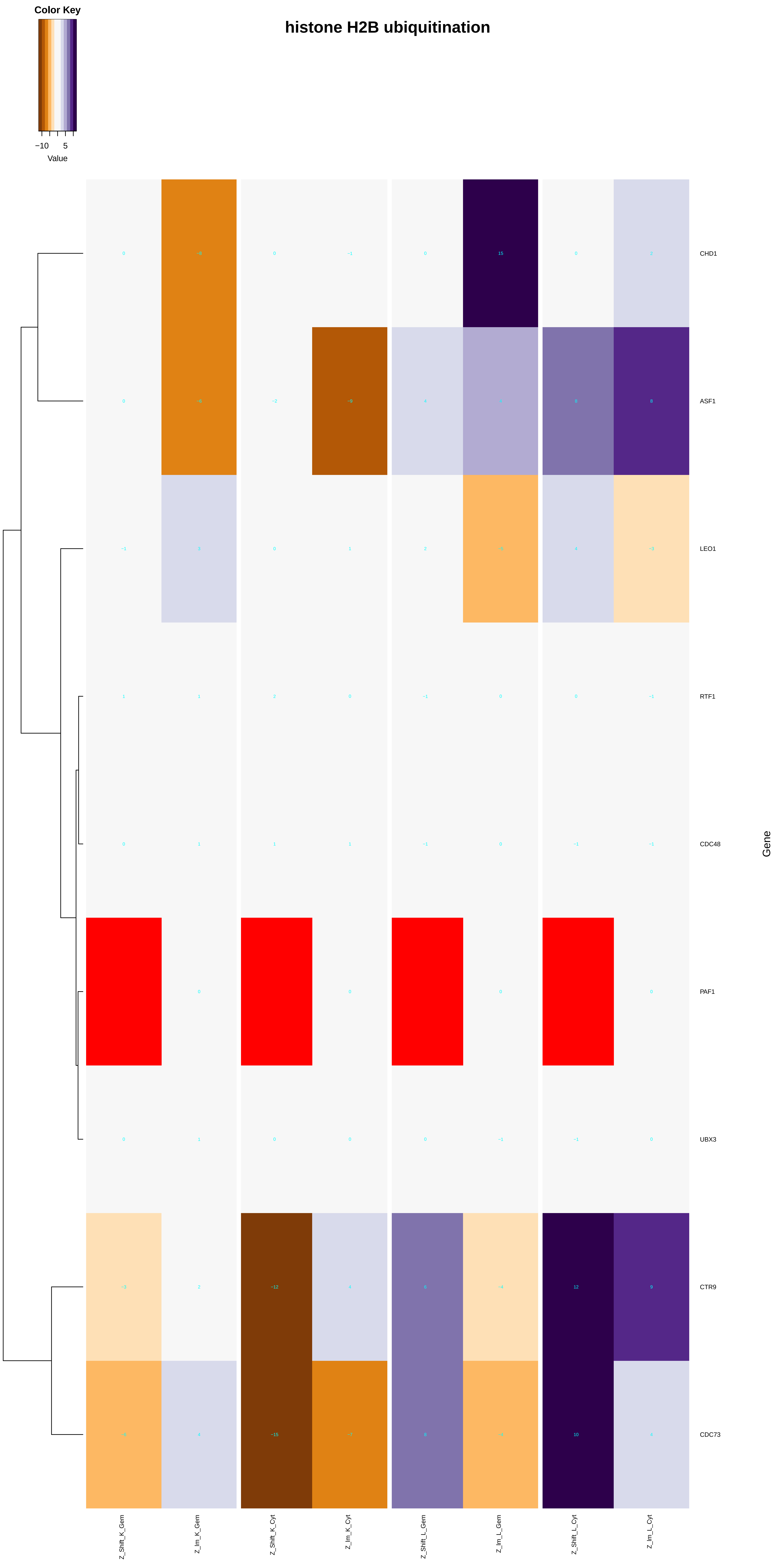

histone H2B ubiquitination
Color Key
−10
5
Value
CHD1
ASF1
LEO1
RTF1
CDC48
PAF1
UBX3
CTR9
CDC73
Gene
Z_Shift_K_Gem
Z_Im_K_Gem
Z_Shift_K_Cyt
Z_Im_K_Cyt
Z_Shift_L_Gem
Z_Im_L_Gem
Z_Shift_L_Cyt
Z_Im_L_Cyt

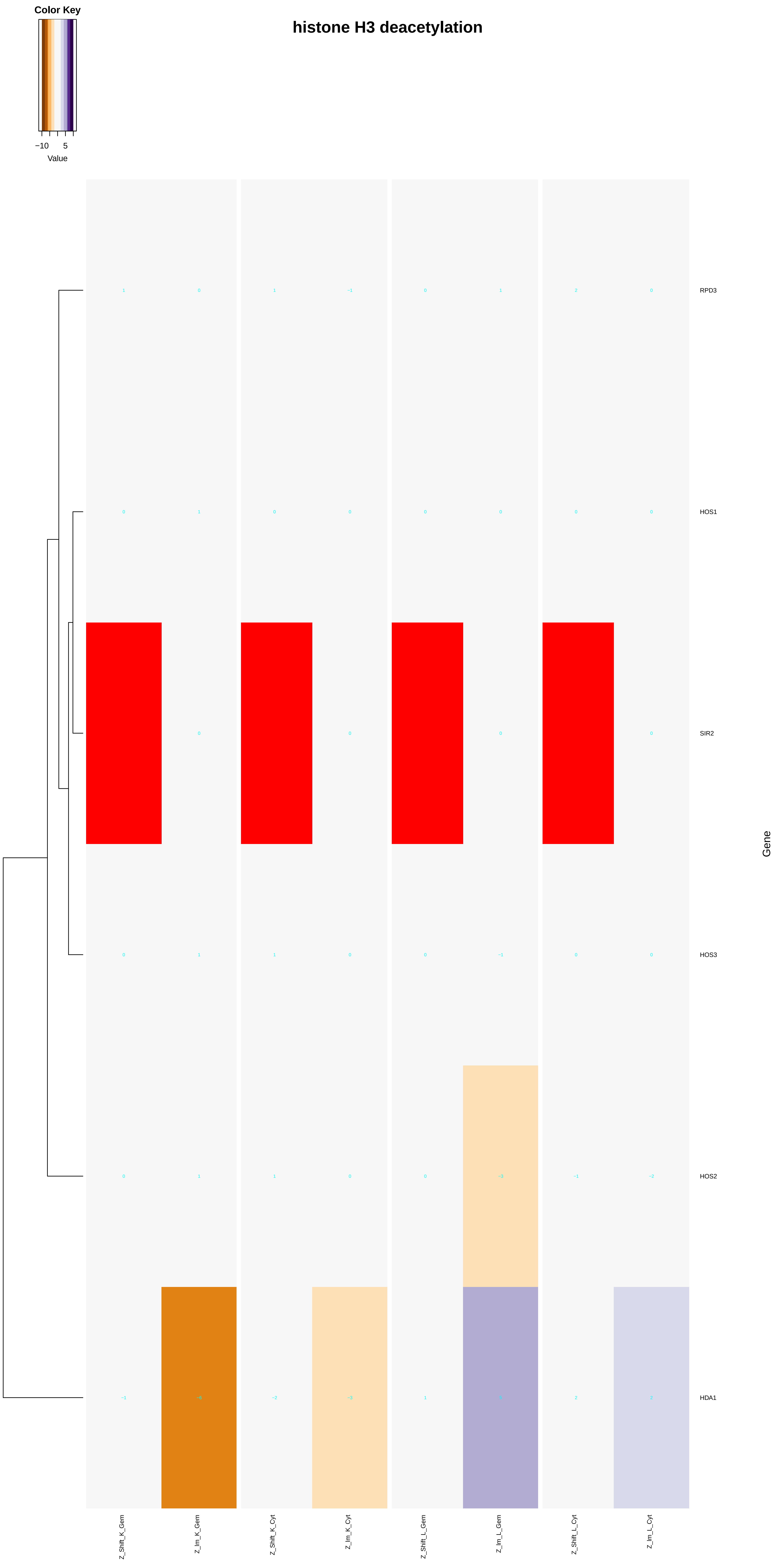

Color Key
−10
5
Value
histone H3 deacetylation
RPD3
HOS1
SIR2
HOS3
HOS2
HDA1
Gene
Z_Shift_K_Gem
Z_Im_K_Gem
Z_Shift_K_Cyt
Z_Im_K_Cyt
Z_Shift_L_Gem
Z_Im_L_Gem
Z_Shift_L_Cyt
Z_Im_L_Cyt

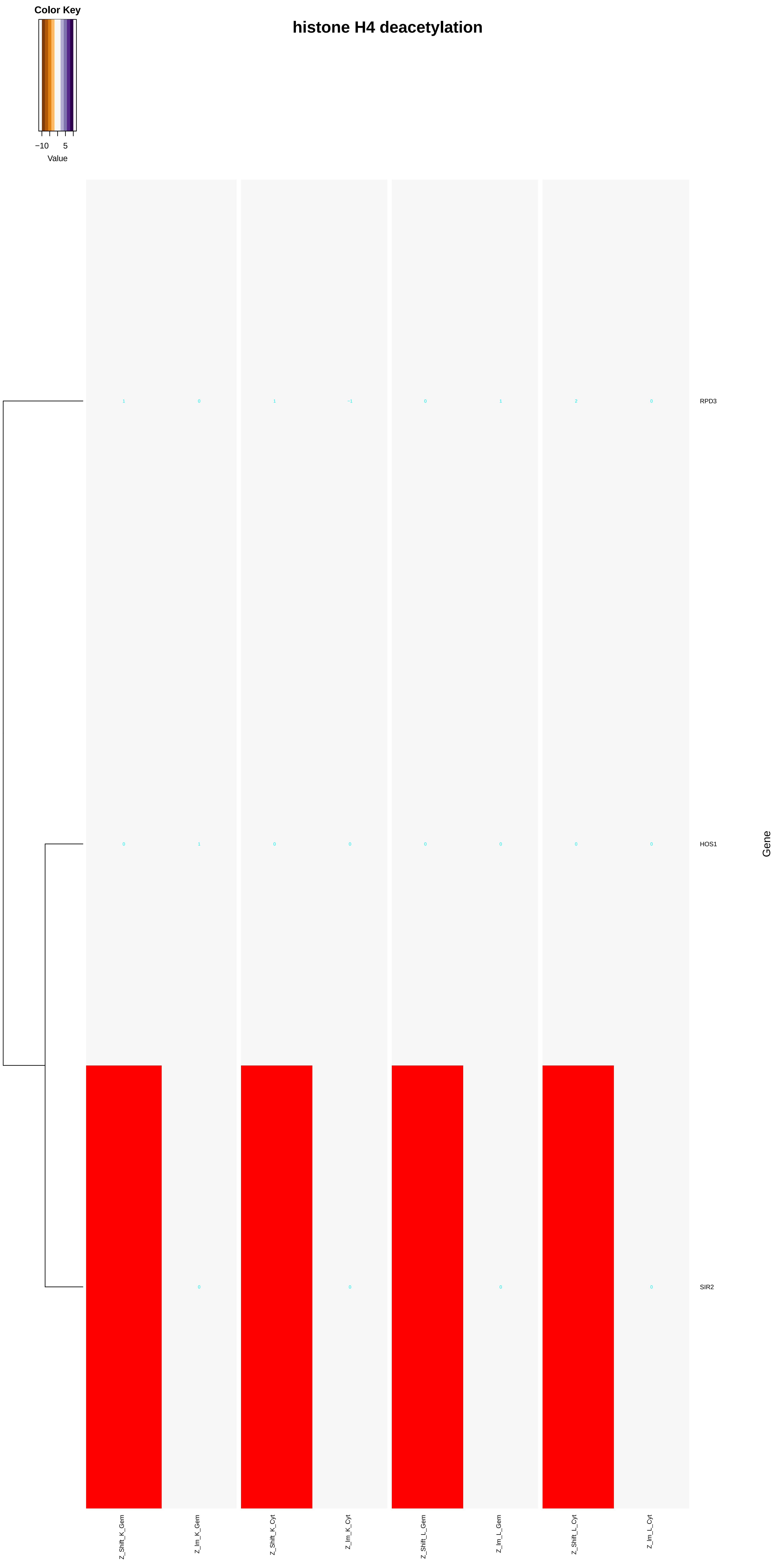
histone H4 deacetylation
Color Key
−10
5
Value
RPD3
HOS1
SIR2
Gene
Z_Shift_K_Gem
Z_Im_K_Gem
Z_Shift_K_Cyt
Z_Im_K_Cyt
Z_Shift_L_Gem
Z_Im_L_Gem
Z_Shift_L_Cyt
Z_Im_L_Cyt

histone lysine demethylation

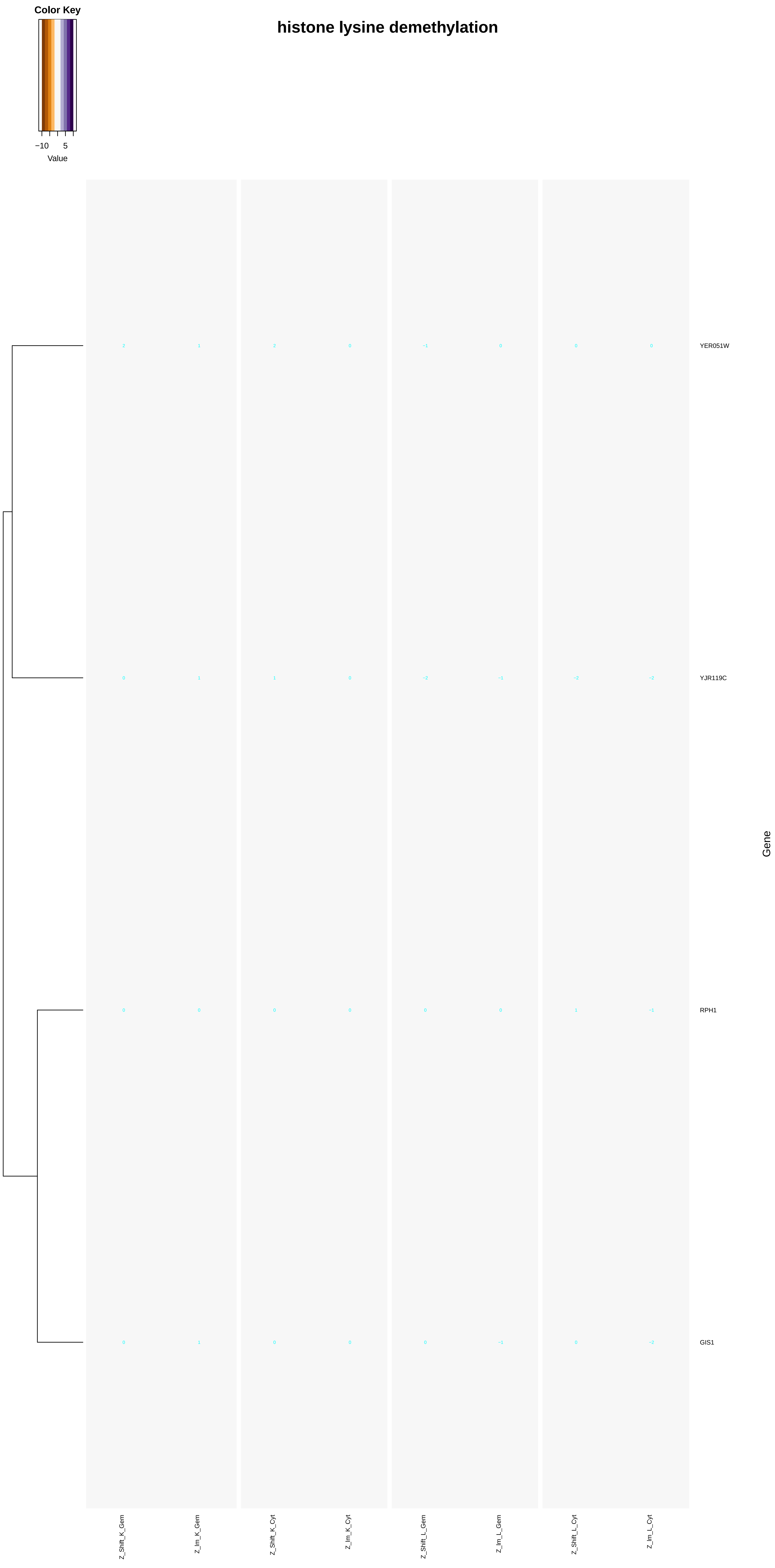

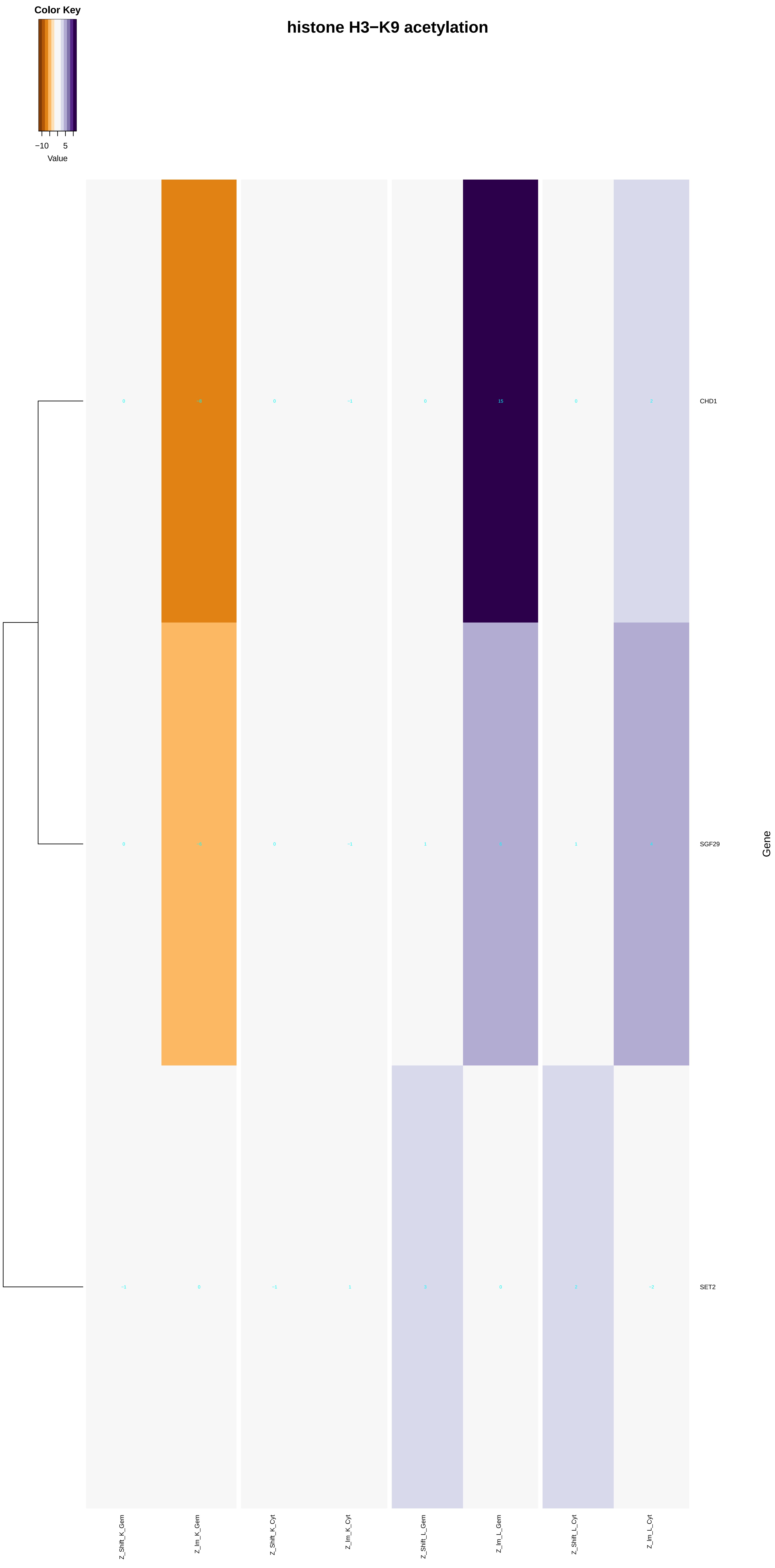

histone H3−K9 acetylation
Color Key
−10
5
Value
CHD1
SGF29
SET2
Gene
Z_Shift_K_Gem
Z_Im_K_Gem
Z_Shift_K_Cyt
Z_Im_K_Cyt
Z_Shift_L_Gem
Z_Im_L_Gem
Z_Shift_L_Cyt
Z_Im_L_Cyt

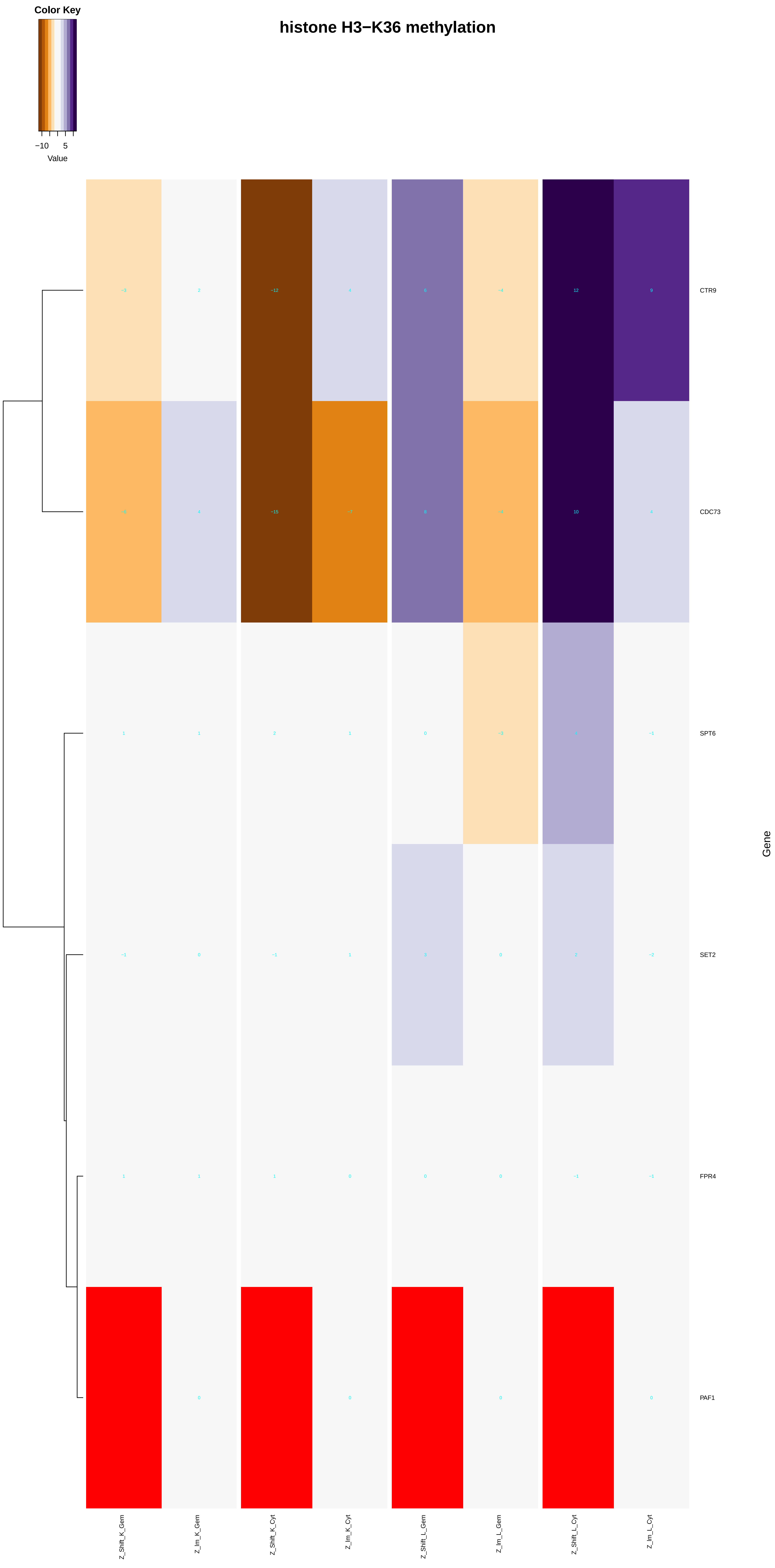
histone H3−K36 methylation
Color Key
−10
5
Value
CTR9
CDC73
SPT6
SET2
FPR4
PAF1
Gene
Z_Shift_K_Gem
Z_Im_K_Gem
Z_Shift_K_Cyt
Z_Im_K_Cyt
Z_Shift_L_Gem
Z_Im_L_Gem
Z_Shift_L_Cyt
Z_Im_L_Cyt

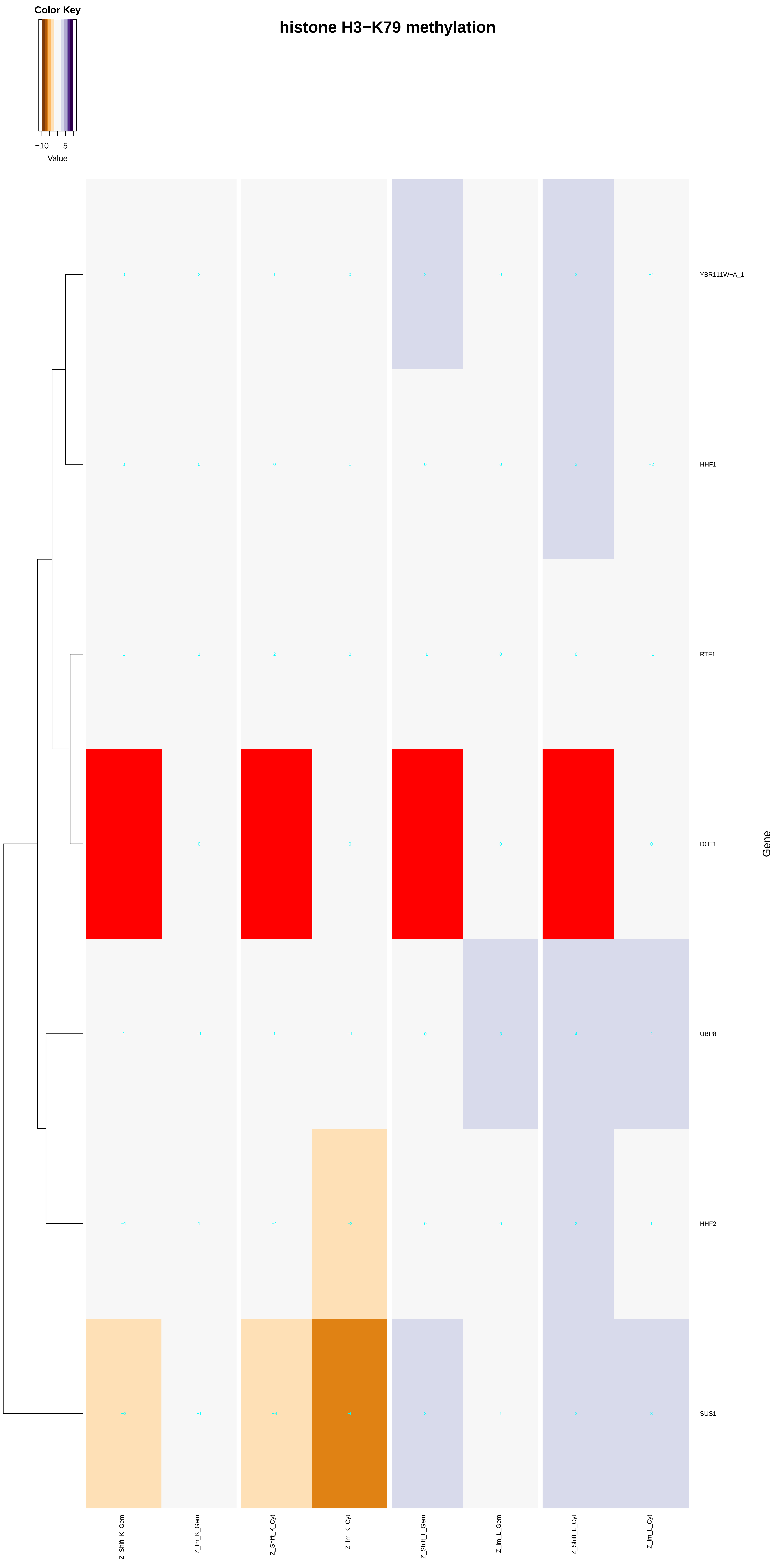
Color Key
−10
5
Value
histone H3−K79 methylation
YBR111W−A_1
HHF1
RTF1
DOT1
UBP8
HHF2
SUS1
Gene
Z_Shift_K_Gem
Z_Im_K_Gem
Z_Shift_K_Cyt
Z_Im_K_Cyt
Z_Shift_L_Gem
Z_Im_L_Gem
Z_Shift_L_Cyt
Z_Im_L_Cyt

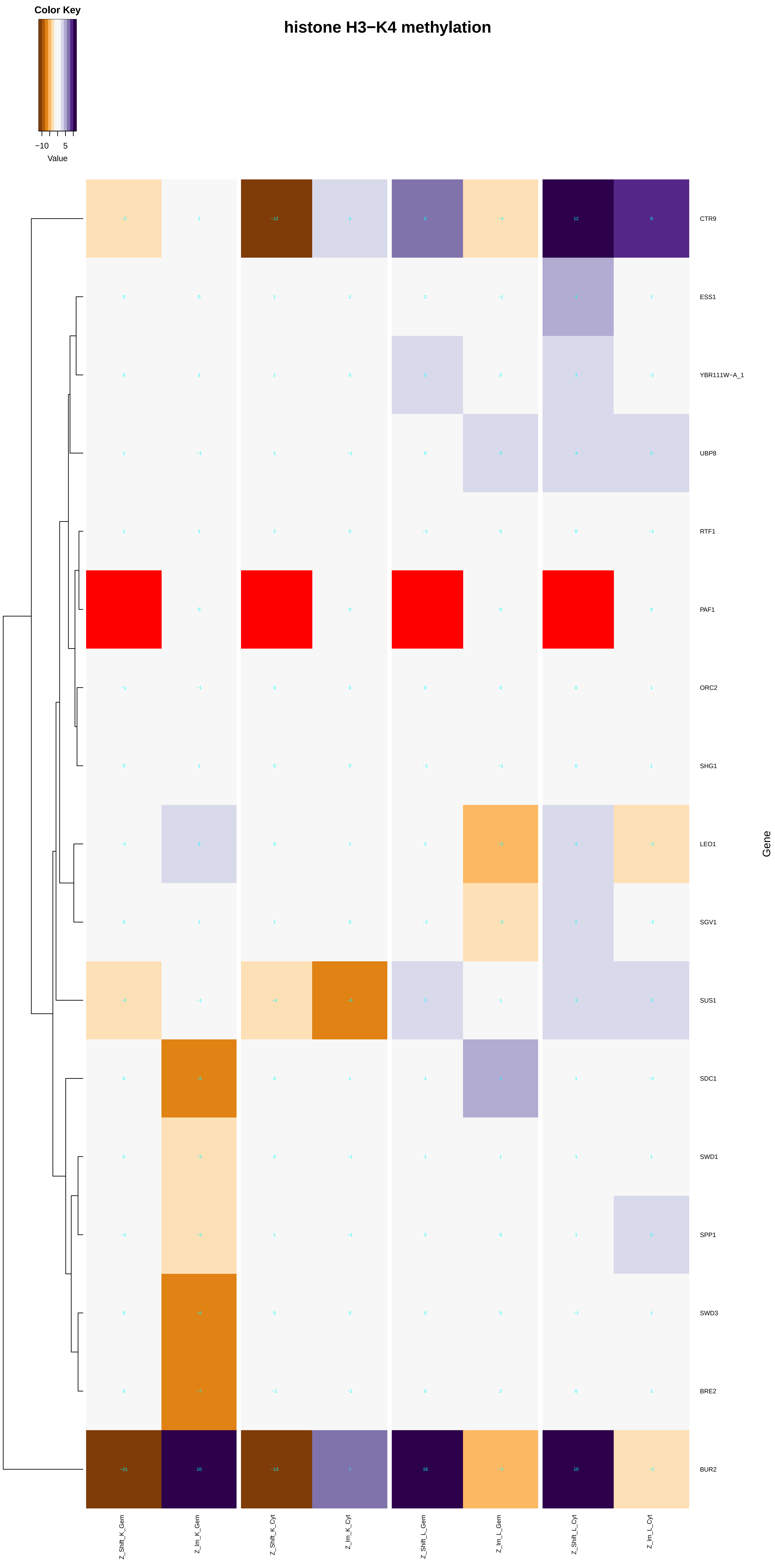

Color Key
−10
5
Value
histone H3−K4 methylation
CTR9
ESS1
YBR111W−A_1
UBP8
RTF1
PAF1
ORC2
SHG1
LEO1
SGV1
SUS1
SDC1
SWD1
SPP1
SWD3
BRE2
BUR2
Gene
Z_Shift_K_Gem
Z_lm_K_Gem
Z_Shift_K_Cyt
Z_lm_K_Cyt
Z_Shift_L_Gem
Z_lm_L_Gem
Z_Shift_L_Cyt
Z_lm_L_Cyt

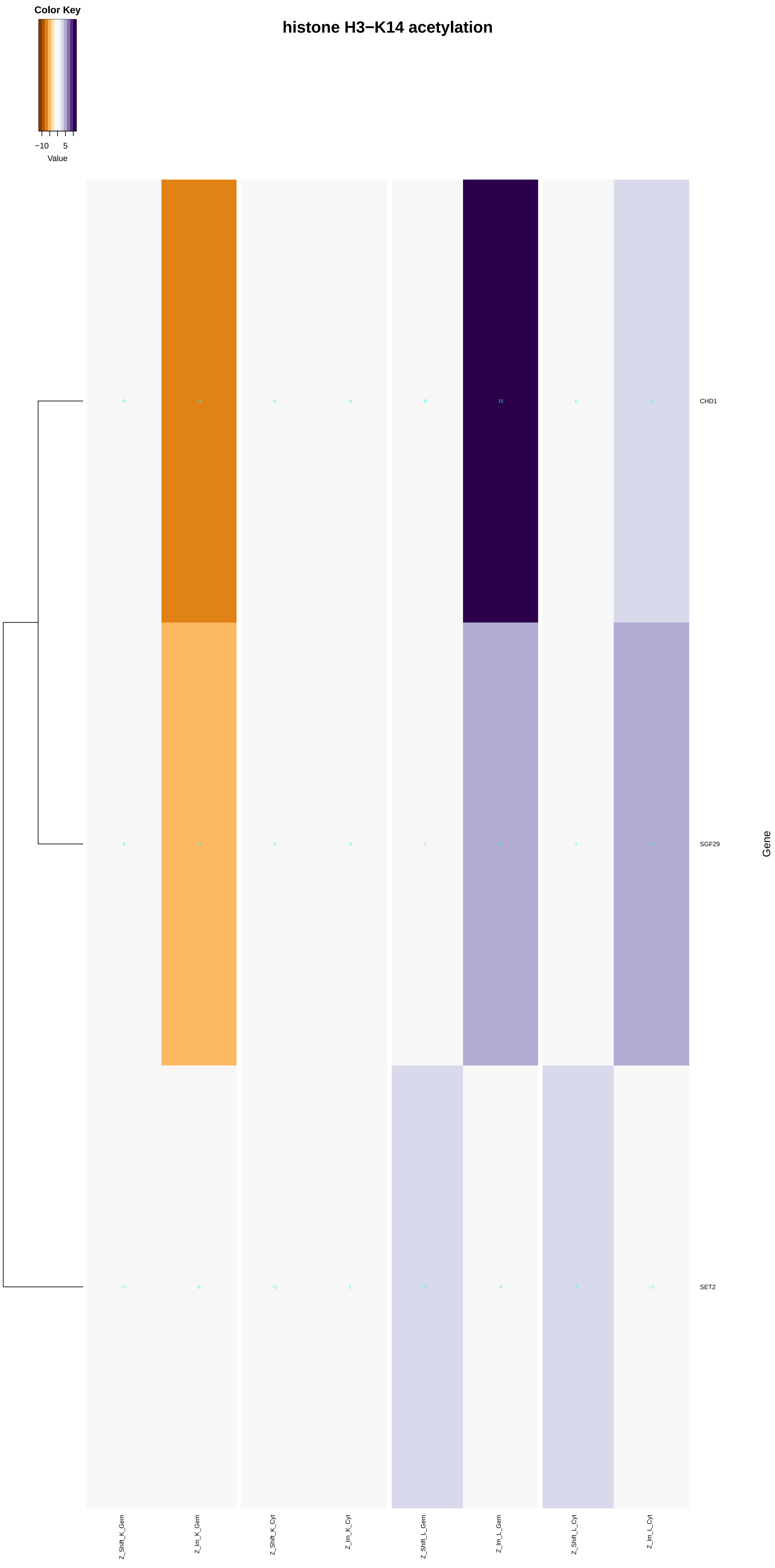

histone H3−K14 acetylation
Color Key
−10
5
Value
CHD1
SGF29
SET2
Gene
Z_Shift_K_Gem
Z_Im_K_Gem
Z_Shift_K_Cyt
Z_Im_K_Cyt
Z_Shift_L_Gem
Z_Im_L_Gem
Z_Shift_L_Cyt
Z_Im_L_Cyt

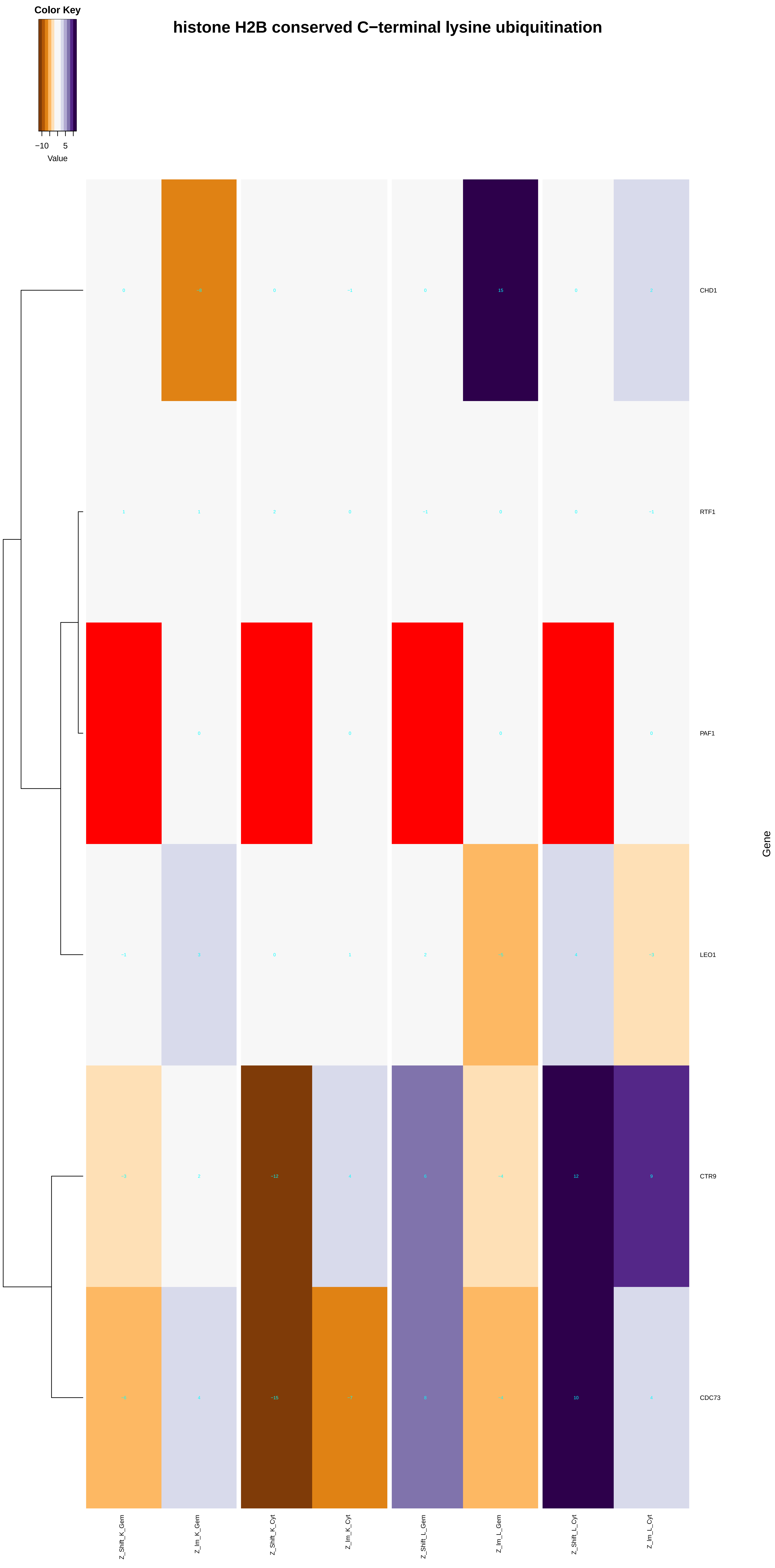

Color Key
−10
5
Value
histone H2B conserved C−terminal lysine ubiquitination
0
−8
0
−1
0
15
0
2
CHD1
1
1
2
0
−1
0
0
−1
RTF1
0
0
0
0
PAF1
−1
3
0
1
2
−5
4
−3
LEO1
−3
2
−12
4
6
−4
12
9
CTR9
−6
4
−15
−7
8
−4
10
4
CDC73
Z_Shift_K_Gem
Z_Im_K_Gem
Z_Shift_K_Cyt
Z_Im_K_Cyt
Z_Shift_L_Gem
Z_Im_L_Gem
Z_Shift_L_Cyt
Z_Im_L_Cyt
Gene

histone H3−K36 demethylation

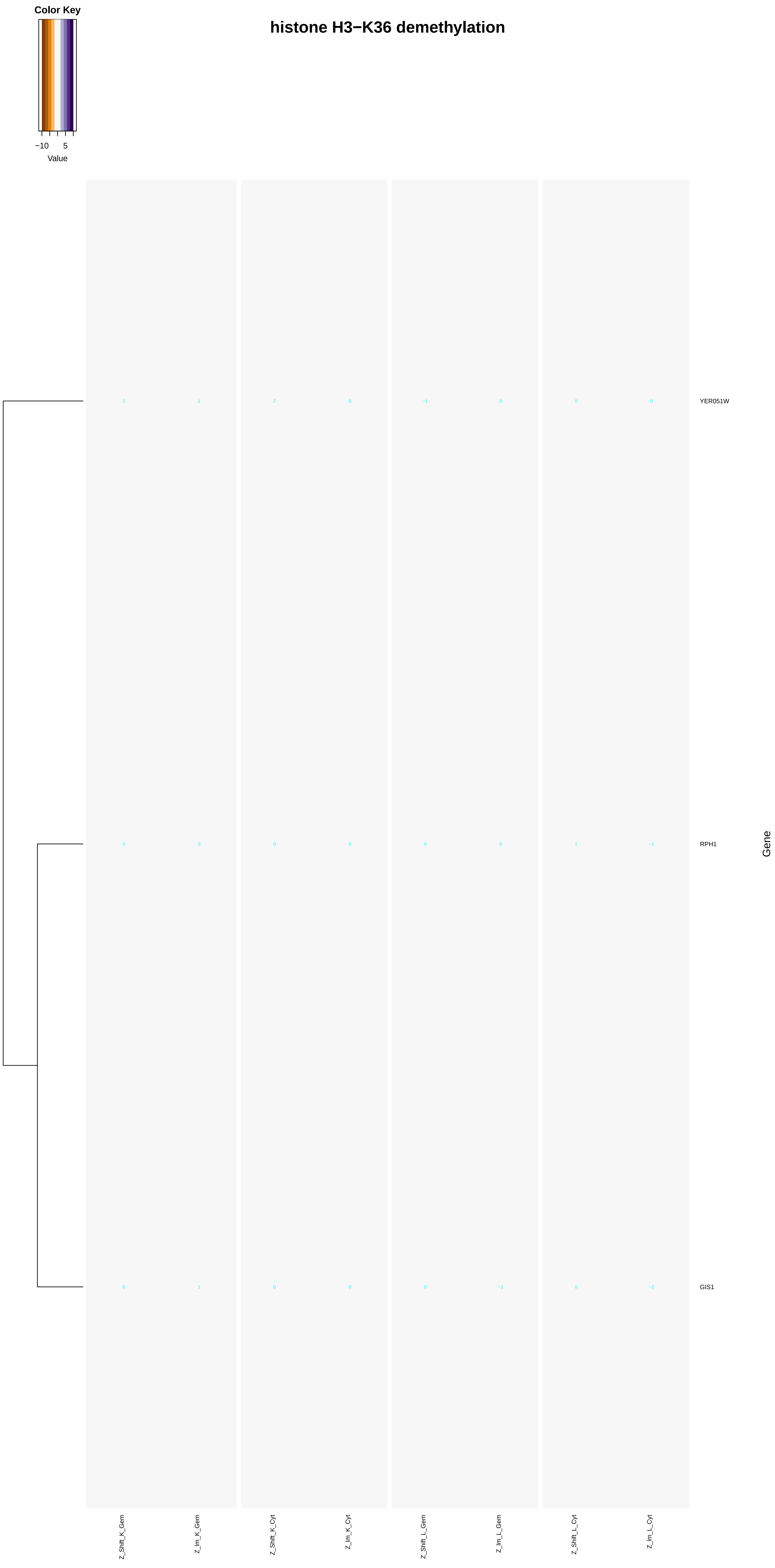

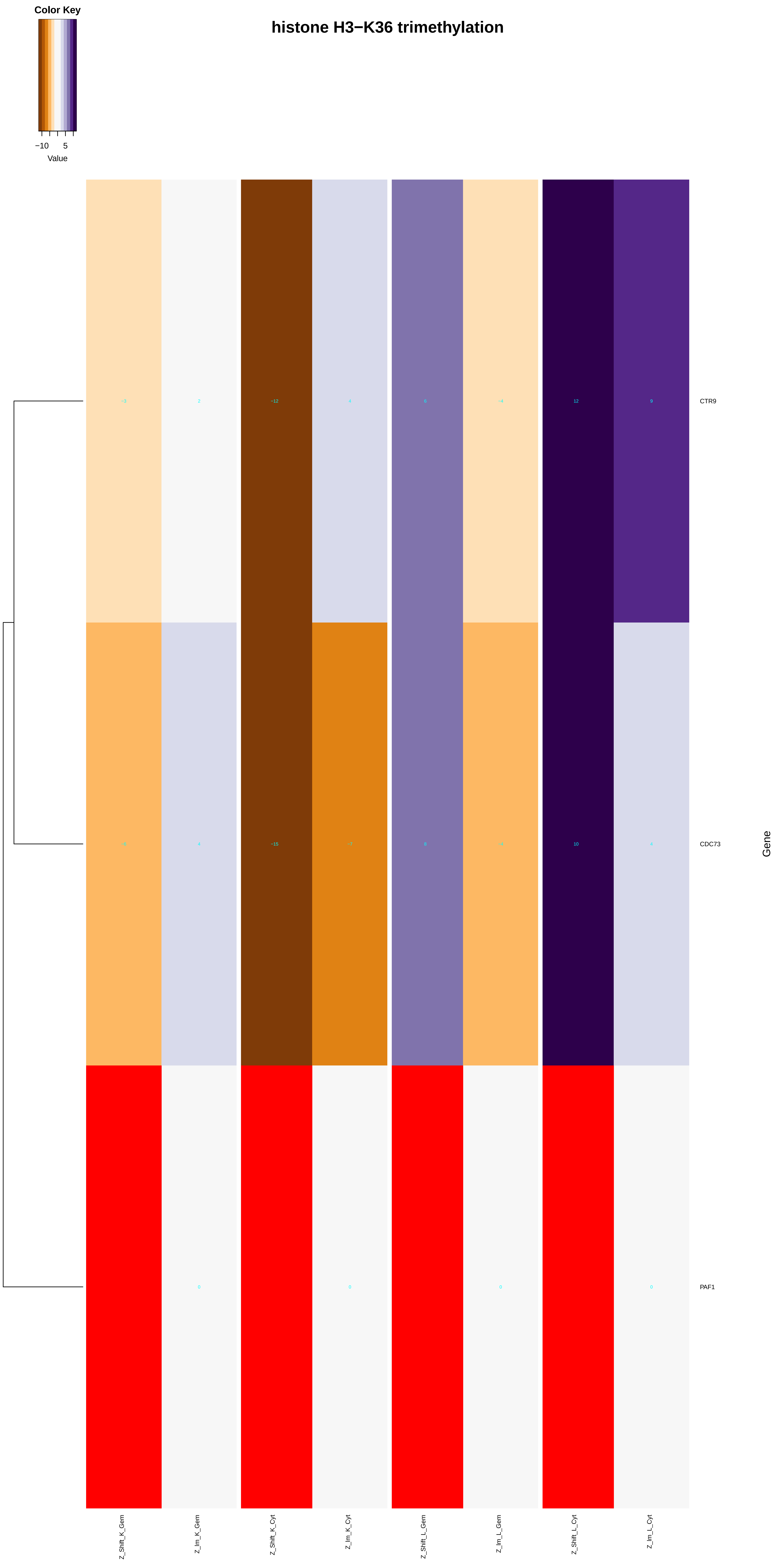
Color Key
−10
5
Value
histone H3−K36 trimethylation
−3
2
−12
4
6
−4
12
9
CTR9
−6
4
−15
−7
8
−4
10
4
CDC73
0
0
0
0
PAF1
Gene
Z_Shift_K_Gem
Z_Im_K_Gem
Z_Shift_K_Cyt
Z_Im_K_Cyt
Z_Shift_L_Gem
Z_Im_L_Gem
Z_Shift_L_Cyt
Z_Im_L_Cyt

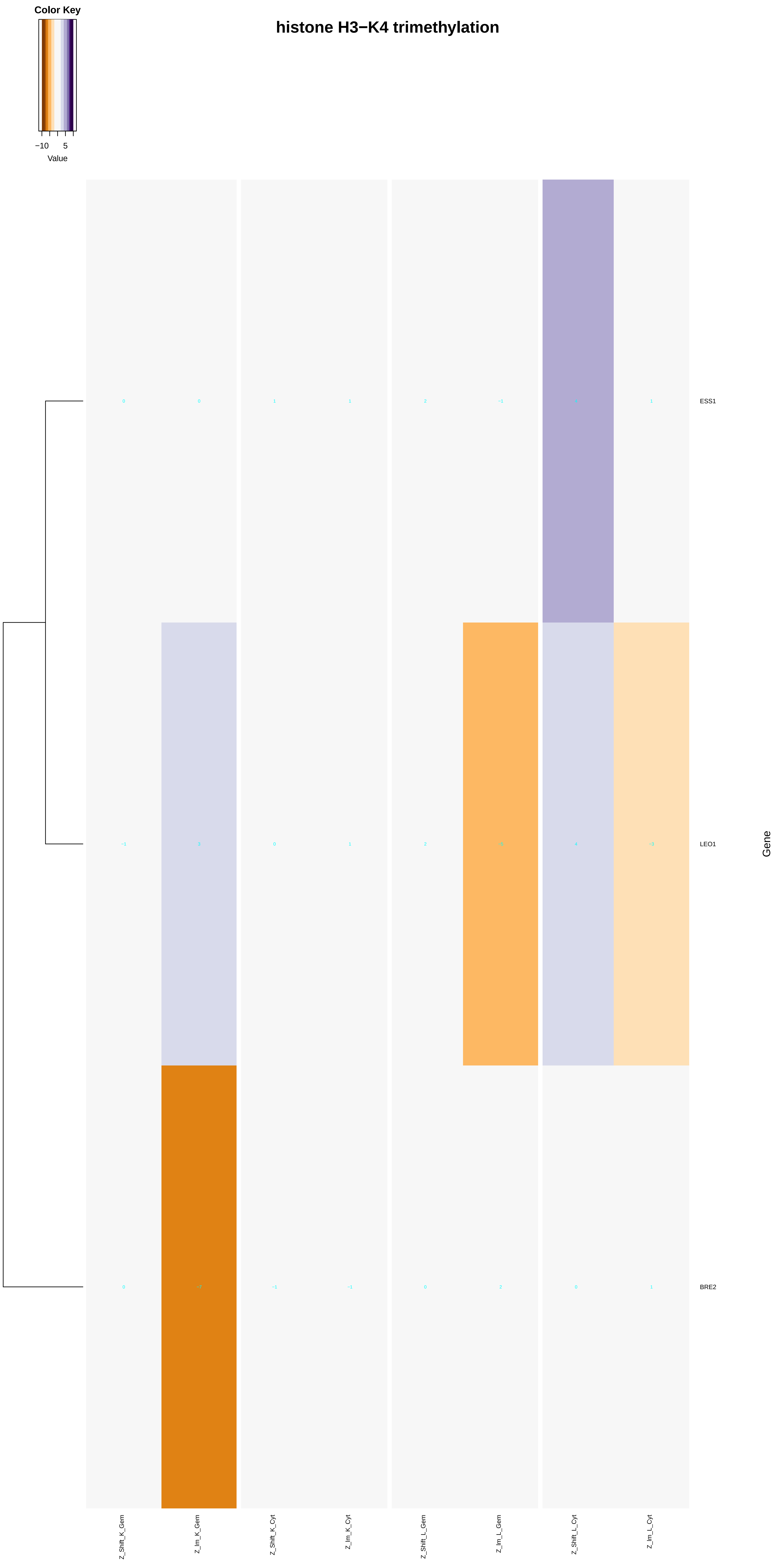

Color Key
−10
5
Value
histone H3−K4 trimethylation
ESS1
LEO1
BRE2
Gene
Z_Shift_K_Gem
Z_Im_K_Gem
Z_Shift_K_Cyt
Z_Im_K_Cyt
Z_Shift_L_Gem
Z_Im_L_Gem
Z_Shift_L_Cyt
Z_Im_L_Cyt
